# Study of Xuanhuang Pill in protecting against alcohol liver disease using ultra-performance liquid chromatography/time-of-flight mass spectrometry and network pharmacology

**DOI:** 10.3389/fendo.2023.1175985

**Published:** 2023-04-04

**Authors:** Xuejie Cui, Maobo Du, Kunhua Wei, Chen Dai, Rachel Y. H. Yang, Bingxue Zhou, Zhaojing Luo, Xiaonan Yang, Yi Yu, Wei Lin, Yi Wu, Yuhong Liu

**Affiliations:** ^1^ College of Pharmacy, Shandong University of Traditional Chinese Medicine, Jinan, China; ^2^ MOE Joint International Research Laboratory of Animal Health and Food Safety, College of Veterinary Medicine, Nanjing Agricultural University, Nanjing, China; ^3^ Institute of Chinese Materia Medica, China Academy of Chinese Medical Sciences, Beijing, China; ^4^ Guangxi Key Laboratory of Medicinal Resources Protection and Genetic Improvement/Guangxi Engineering Research Center of TCM Resource Intelligent Creation, Guangxi Botanical Garden of Medicinal Plants, Nanning, China; ^5^ College of Life Sciences, Nanjing Agricultural University, Nanjing, China; ^6^ La Jolla Country Day School, La Jolla, CA, United States; ^7^ Department of Anesthesiology, Affiliated Stomatological Hospital, Nanjing Medical University, Nanjing, Jiangsu, China

**Keywords:** alcohol liver disease, chemical components, molecular docking, network pharmacology, Proteomics

## Abstract

**Introduction:**

Xuanhuang Pill (XHP) is a traditional Chinese medicine oral formula composed of 10 herbs. This study aims to verify the hepatoprotective activity of XHP and explain its possible mechanism.

**Methods:**

The hepatoprotective activity of XHP was evaluated by constructing a mouse model of alcoholic liver disease, and the mechanism of XHP was preliminarily explained by utilizing ultra-performance liquid chromatography/time-of-flight mass spectrometry (UPLC-QTOF/MS), proteomics and network pharmacology.

**Results:**

The current study demonstrated that treatment with XHP ameliorated acute alcohol-induced liver injury in mice by significantly reducing alanine aminotransferase (ALT) and aspartate aminotransferase (AST) levels and triglycerides (TGs) and malondialdehyde (MDA) content. Remarkably, treatment also increased superoxide dismutase (SOD) activity and glutathione (GSH) content. UPLC-QTOF/MS, 199 compounds were identified as within the make-up of the XHP. Network pharmacology analysis showed that 103 targets regulated by 163 chemical components may play an important role in the protective liver effect mediated by XHP. Kyoto Encyclopedia of Genes and Genomes (KEGG) enrichment analysis suggest that the HIF-1, FoxO, PI3K-Akt, insulin, and thyroid hormone signaling pathways are key modulators of XHP’s effects. Finally, eight key targets including Mapk1, Mapk3, Akt1, Map2k1, Pik3ca, Pik3cg, Raf1, and Prkca were verified by molecular docking and proteomics analysis, which provide insight into the hepatoprotective effect observed with XHP treatment.

**Conclusion:**

In summary, these results improved upon knowledge of the chemical composition and the potential mechanisms of hepatoprotective action of oral XHP treatment, providing foundational support for this formulation as a viable therapeutic option for alcoholic liver disease.

## 1 Introduction

Excessive drinking is an important public health problem in the world and a major risk factor for high morbidity and mortality from alcohol liver disease (ALD), which is the second most common liver disease (1, 2). ALD is a complex syndrome that consists of several stages including mild alcoholic liver disease, alcoholic fatty liver disease, alcoholic hepatitis, alcoholic liver fibrosis, and alcoholic cirrhosis (3, 4). Because ALD is an advanced and incurable progressive disease, early prevention and treatment can attenuate the development of irreversible lesions (5).

In China, traditional Chinese medicine (TCM) to prevent and treat ALD has historically been a common treatment regimen. For example, *Bupleurum chinense* DC. (*B. chinense*) and *Scutellaria baicalensis* Georgi (*S. baicalensis*) are routinely used as modern clinical herbal treatments for liver disease (6). Additionally, *Ephedra sinica* Stapf (*E. sinica*), *Cinnamomum cassia* (L.) J.Presl (*C. cassia*), and *Smilax glabra* Roxb. (*S. glabra*) have been shown to mediate benefits within the liver. The rest of the herbs are shown to strengthen and eliminate toxins (7–12). On the basis of summarizing various classical liver-protecting prescriptions, this study combines commonly used TCM to make a novel treatment, called the XHP. While, anecdotally, this XHP combination had a positive feedback within the community for patients suffering from ALD, scientific evidence regarding the effect of XHP as a treatment for ALD lacking the mechanism of action for potential liver benefits is unclear.

The recent rise in network pharmacological approaches provides an effective opportunity for studying the effects of a multi-component and multi-target therapy, such as that of complex TCM formulations (13, 14). However, traditional network pharmacology methods are typically established by querying chemical components present in established databases, but the exact composition of many TCM therapies are not well understood.

However, advancements within modern high-resolution mass spectrometry and bioinformatics provide the opportunity for identifying the chemical formulation of previously enigmatic TCM therapies. Specifically, ultra-performance liquid chromatography/time-of-flight mass spectrometry (UPLC-QTOF/MS) coupled with UNIFI software offers the advantage of reliably identifying specific compounds within commonly used TCM.

In the present study, the above techniques were utilized to study the hepatoprotective effect and mechanism of XHP in the setting of ALD. The comprehensive chemical profile of XHP was identified with UPLC-QTOF/MS together with UNIFI software. A mouse model of acute alcoholic liver injury was employed to evaluate the hepatoprotective efficacy of XHP. Network pharmacology analysis predicted potential XHP targets, which were confirmed with proteomics and molecular docking experiments.

## 2 Materials and methods

### 2.1 Materials and reagents

XHP was provided by Beijing Shengai Zichen Pharmaceutical Technology Co., Ltd. (Beijing, China). Bifendiester Dropping Pills were purchased from Peking Union Medical College (Beijing, China). Anhydrous ethanol was purchased from Guangdong Guanghua Technology Co., Ltd. (Guangzhou China). Alanine aminotransferase (ALT), aspartate aminotransferase (AST), triglyceride (TG), malondialdehyde (MDA), glutathione (GSH), and superoxide dismutase (SOD) kits were purchased from Nanjing Jiancheng Bioengineering Institute (Nanjing, China). Acetonitrile and methanol were purchased from Merck Drugs & Biotechnology (Darmstadt, GER). Formic acid was purchased from Sigma Chemical Co. (St. Louis, MO, USA). Ultrapure water was prepared by Merck Milli-Q (Darmstadt, GER).

### 2.2 Animal experiment design

A total 36 male C57BL/6 mice (6 weeks old, body weight 20 ± 2 g) were purchased from the Center for Comparative Medicine, Yangzhou University (Jiangsu, China) and housed in cages at room temperature (22 ± 2°C) with 12 h/12 h light/dark cycle. Before the start of the formal experiment, the concentration of XHP from 5 mg/ml to 1 g/ml was screened by *in vitro* experiments. Finally, 60, 120, and 240 mg/ml were selected as the drug concentrations of animal experiments. After 1 week of adaptive feeding, mice were randomly divided into six groups (n = 6 per group): normal group, model group, 15 mg/ml bifendate group, 600 mg/kg XHP (XHP-L) group, 1,200 mg/kg XHP (XHP-M) group, and 2,400 mg/kg XHP (XHP-H) group. Bifendate group was intragastrically administered with 15 mg/ml bifendate at 10 ml/kg/day, and each administration group was also intragastrically administered with the corresponding concentration of XHP at 10 ml/kg/day for a total of 15 days. After 6 h of the last administration, all groups except the normal group were exposed to gastric irrigation with alcohol (14 ml/kg, 50% alcohol) and then fasted without water. Animal studies were performed following the recommendations in the Guidelines for the Care and Use of Laboratory Animals and relevant Chinese laws and regulations, which was approved by the Animal Ethics Committee of Nanjing Agricultural University.

Blood was collected utilizing the retro-orbital method with anesthesia and centrifuged to obtain serum. Liver tissues was collected and stored at −80°CC for further experiments.

### 2.3 XHP preparation

XHP was formulated by weighing out 9 g *B. chinense*, 3 g *S. baicalensis*, 3 g *E. sinica*, 6 g *C. cassia*, 9 g *R. glutinosa*, 6 g *S. glabra*, 30 g *L. chinense*, 6 g *P. ginseng*, and 6 g *G. uralensis* adding ultra-pure water at a solid–liquid ratio of 1:20. The XHP extract was obtained by ultrasonic extraction for 1 h, and the supernatant was collected by centrifugation (4,000 rpm, 15 min).

### 2.4 UPLC-QTOF/MS analysis

The XHP formulation was analyzed on a Waters ACQUITY UPLCTM liquid chromatograph (MA, USA) equipped with an UPLC column (BEH C18 2.1 mm×100 mm, 1.8 μm). The mobile phases consisted of (A) 0.1% formic acid aqueous solution and (B) acetonitrile solution. The gradient elution was optimized as follows: 0–1 min, 2% B; 1–22 min, 2%–95% B; 22–26.4 min, 95% B; 26.4–26.5 min, 95%–2% B; and 26.5–30 min, 2% B. The flowrate was 0.3 ml/min, the column temperature was 40°C, the autosampler temperature was 10°C, and the injection volume was 5 μl.

Mass spectrometric data were obtained by a Waters Xevo G2-XS QTof mass spectrometer (MA, USA). The electrospray ion source was in positive and negative ion scanning mode, scanning range of m/z 50–1,000, scanning time of 0.5 s, capillary voltage of 3 kV, cone voltage of 30 V, and MSE collision energy low energy set to 6 V. The high energy was set as 20–45 V, the cone gas flowrate was 50 L/h, the ion source temperature was 100°C, the desolvation temperature was 400°C, and the desolvation gas flowrate was 600 L/h.

Based on the screening conditions of oral bioavailability ≥30 and drug-likeness >0.18, the TCMSP database (https://old.tcmsp-e.com/tcmsp.php **)** (15) was searched to obtain the chemical composition information of each single herb in XHP, including name, molecular formula, structural formula (.mol format), to establish the chemical composition database of XHP. This database was then imported into the UNIFI 2.0 software.

### 2.5 Network pharmacology analysis

#### 2.5.1 Targets of components and ALD

The identified XHP components were imported into the “Phammapper” (http://www.lilab-ecust.cn/pharmmapper/) and “Swiss Target Prediction” (http://www.swisstargetprediction.ch/) databases, and targets were identified using the probability >0 as the screening condition.

ALD targets were obtained from GeneCards database using “alcohol liver disease, ALD” as keywords. Targets and the active components of XHP were overlapped with the ALD target results to identify XHP targets enriched in the ALD pathways.

#### 2.5.2 Bioinformatics analysis

Functional protein association networks (STRING, https://cn.string-gb.org/) (16) were used to obtain the protein–protein interaction (PPI) data and Kyoto Encyclopedia of Genes and Genomes (KEGG) enrichment analysis.

#### 2.5.3 Construction of networks

The herb–component–target–pathway network was created using Cytoscape 3.8.2. Nodes in networks represented herbs, targets, and pathways, and edges indicated interactions between herbs and components, components and targets, or targets and pathways. For the topological properties, the degree was calculated using Analyze Network tools to indicate the importance of nodes.

### 2.6 Proteomics analysis

Total protein from collected liver samples was extracted with radioimmunoprecipitation assay (RIPA) buffer with protease inhibitor and quantified using the bicinchoninic acid (BCA) method (Beyotime Biotechnology). A total of 200 μg protein was added to a final concentration of 10 mM dithiothreitol (DTT) 35°CC for 1.5 h, then restored to room temperature. Iodacetamide with final concentration of 20 mM was added at room temperature for 40 min. Samples were diluted fourfold with 25 mM ammonium bicarbonate. Trypsin was added at 50:1 ratio of protein to trypsin at 37°CC overnight. Digested peptides were washed with 200 μl 60% acetonitrile (ACN), centrifuge at 5,000 relative centrifugal field (RCF) for 2 min. A total of 200 μl 0.2% formic acid was washed once, centrifuged at 5,000 RCF for 2 min. The sample was added and then centrifuged at 5,000 RCF for 2 min. The column was next washed with 200 μl 0.2% formic acid, centrifuged at RCF for 2 min. The elute was replaced with 60% ACN, and the eluent was collected and lyophilized.

Mobile phase solution A (100% mass spectrometry water, 0.1% FA) and liquid B (80% ACN, 0.1% FA) were prepared. Tryptic peptides were dissolved in A solution and injected for liquid quality detection. Chromatographic separation was carried out on the analytical column (Acclaim PepMap^®^ RSLC, C18, 75 μm × 15 cm, 3 μm, 100 Å, Thermo Scientific) using a linear gradient of 3%–35% buffer B at a flowrate of 0.3 μl/min over 70 min. The compensation voltage (CV) of FAIMS was −45 and −65 V. The mass spectrometer was operated in data-dependent analysis (DDA) mode with dynamic exclusion of 30 s and full-scan MS spectra (m/z 350–1,500) with resolution of 60,000 (m/z 200), followed by fragmentation of the most intense ions within 1 s cycle time with high energy collisional dissociation (HCD), normalized collision energy (NCE) of 30.0, and resolution of 15,000 (m/z 200) in MS/MS scans. Raw data were imported to Proteome Discoverer (Version: 2.4, Thermo Scientific) for protein identification.

### 2.7 Molecular docking analysis

In this study, we aimed to ascertain the interaction between targets and their related compound to obtain the crucial compounds. Hence, key targets and related compounds were chosen for verification of molecular docking. The Schrödinger (ver. 3.3) platform was used to predict compound–target molecular docking. Crystal structures of the targets were obtained from the Protein Data Bank website (https://www.rcsb.org/).

### 2.8 Statistical analysis

All data were expressed as mean ± relative standard deviation. Differences between groups were analyzed using two-way ANOVA. The second was to apply the least significant difference *post-hoc* test. GraphPad Prism 8.0 software were used for all analyses. A threshold of p < 0.05 indicated statistical significance, p < 0.01 indicated a very statistically significant difference, and p > 0.05 meant that no significant difference was observed.

## 3 Results and discussion

### 3.1 Effects of XHP for ALD

**Figure 1:**
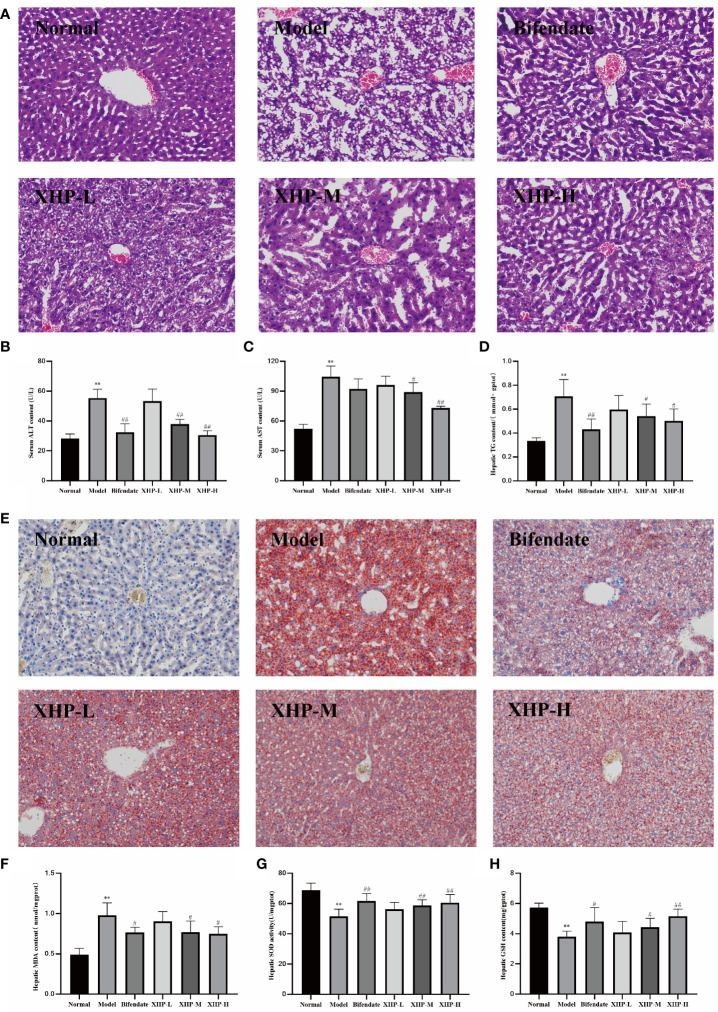
XHP alleviated lipid accumulation, oxidative stress, and degree of damage in the liver of alcohol induced mice. **(A)** Representative images of H&E (200×) staining of liver sections. **(B, C)** Serum ALT and AST contents. **(D)** Hepatic TG contents. **(E)** Representative images of Oil-red O (200×) staining of liver sections. **(F)** Hepatic MDA contents. **(G)** Hepatic SOD activities. **(H)** Hepatic GSH contents. Compared with the normal group, ^**^ *p* < 0.01; compared with the model group, ^#^ *p* < 0.05, ^##^ *p* < 0.01.

Histological analysis *via* HE staining (**Figure 1A**) demonstrated that livers from mice in the healthy control group exhibited normal hepatic lobe structure and typical hepatic cord arrangement. As expected, analysis of liver cells from mice in the alcohol-only model group exhibited moderate flaky edema, disordered hepatic cord arrangement, and swelled hepatocytes surrounding the central vein, and vesicular steatosis was observed in the cytoplasm. In mice treated with alcohol, XHP exposure dose-dependently ameliorated these pathological markers. The contents of ALT and AST in serum were key biological indicators for evaluating liver function, which could directly reflect the degree of liver damage (17). Alcohol induced a significant increase in serum ALT and AST, with the AST content almost twofold higher than ALT, which is a typical feature of alcoholic liver disease clinically. Treating mice with alcohol liver injury with either the XHP-M and XHP-H dose significantly reduced serum ALT and AST levels (**Figures 1B, C**).

Excessive ethanol intake could induce fat synthase activity and accumulate a large amount of triglycerides, which in turn would lead to abnormal blood circulation in the liver, increased portal pressure, and accelerated deterioration of ALD (18, 19). As shown in **Figure 1D**, the triglyceride content of the alcohol-control group was twofold higher relative to healthy controls. Both the XHP-M and XHP-H doses significantly reduced TG levels in the liver (p < 0.05), suggesting that XHP treatment regulates TG liver storage. Oil red O staining (**Figure 1E**) further confirmed this observation, as a large number of lipid droplets were apparent in alcohol-only samples, and XHP attenuated this response in a dose-dependent manner.

Oxidative stress is an important mechanism underlying the progression of ALD (20). SOD activity and MDA and GSH content are reliable measures of antioxidant capacity (21–23). XHP treatment in mice experiencing alcohol-induced liver injury dose-dependently reduced levels of oxidative stress as the greatest benefits with regard to MDA levels (**Figure 1F**), SOD activity (**Figure 1G**), and GSH levels (**Figure 1H**) returned to levels observed in healthy controls with XHP-H. Taken together, the hepatoprotective mechanism of XHP may be related to enhancing the liver’s ability to resist lipid peroxidation and scavenge oxygen free radicals.

### 3.2 Identification of chemical constituents in XHP

The constructed XHP library contained a total of 1,501 chemical components, 363 from *E. sinica*, 349 from *B. chinense*, 280 from *G. uralensis*, 220 from *C. cassia*, 190 from *P. ginseng*, 188 from *L. chinense*, 143 from *S. baicalensis*, 76 from *R. glutinosa*, 55 from *A. macrocephala*, and 34 from *S. glabra*.

**Figure 2:**
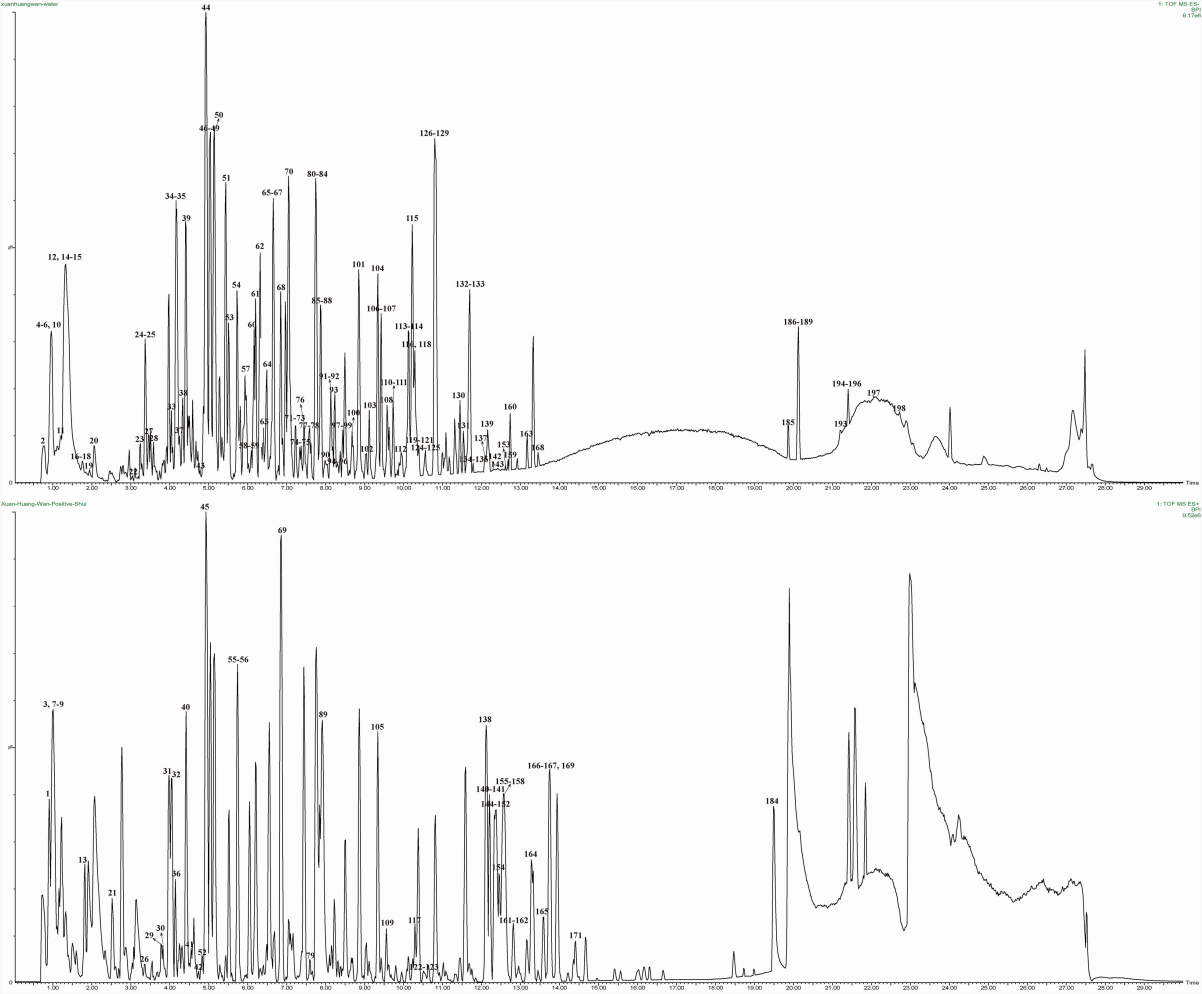
BPI chromatograms of XHP obtained by UPLC-QTOF/MS. The top picture was negative ions, and the bottom picture was positive ions.

The mass spectrometry data collected in positive and negative ion mode were automatically referenced to the UNIFI database. The adducts of +H and +Na were selected to identify target components in positive mode, and the adducts of –H and +HCOO were selected in negative mode. Compounds with a deviation ± 2 mDa were selected, identified, and verified, and final results were obtained. A total of 199 compounds were screened from the XHP database, and the base peak ion (BPI) chromatogram is shown in **Figure 2**. Specific structures are shown in **Supplementary Figure S1**, including 43 flavonoids, 25 esters, 34 organic acids, 16 saponins, 16 glycosides, 11 ketones, 9 aldehydes, 5 coumarins, 5 alkaloids, and 35 others. The retention times, molecular formulas, and secondary fragments of the 199 chemical components are provided in **Supplementary Table S1**.

### 3.3 Structure characterization of the compounds in XHP

#### 3.3.1 Flavonoids

Flavonoids are divided into several subclasses, such as flavanols, flavanones, anthocyanins, isoflavones, and flavonoids. The backbone of diphenylpropane consists of two benzene rings (A and B), and the pyran ring connecting the A ring connects the two benzene rings. The flavonoids are dominated by the fragmentation of small neutral molecules such as CO, CO_2_, and H_2_O, and the cleavage of Retro Diels–Alder reaction (RDA) and sugar groups under mass spectrometry conditions.

**Figure 3:**
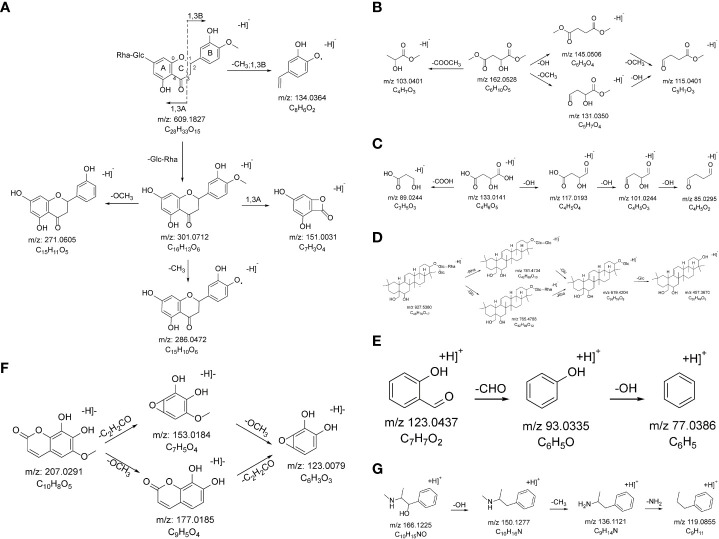
The probable MS fragmentation process of Hesperidin **(A)**, Dimethyl L-malate **(B)**, (2S)-2-Hydroxybutanedioic acid **(C)**, Saikosaponin F **(D)**, Salicylaldehyde **(E)**, Fraxetin **(F)**, and Pseudoephedrine **(G)**.

A total of 43 flavonoids were identified as part of the XHP formulation, 21 of which were *G. uralensis*, 12 *S. baicalensis*, 12 *E. sinica*, 9 *B. chinense*, 3 *L. chinense*, and 1 chemical component belonging to *P. ginseng*. Taking compound 70 as an example, in negative ion mode, its quasi-molecular ion peak is m/z 609.1827 [M-H]^−^, and the elemental composition of this compound is C_28_H_34_O_15_. The parent ion is broken off in 1,3 B mode, and a molecule of CH_3_ is removed to obtain m/z 134.0365 [M-H-CH_3_-Glc-Rha-C_5_H_3_O_3_]^−^. Glucose and rhamnose are removed from the parent ion to obtain m/z 301.0712 [M-H-Glc-Rha]^−^, which was broken in the 1,3A mode in the next step to obtain m/z 151.0031 [M-H-Glc-Rha-C_9_H_10_O_2_]^−^. One molecule of CH_3_ and one molecule of OCH_3_ were removed from the m/z 301.0712 [M-H-Glc-Rha]^−^, respectively, to obtain m/z 286.0472 [M-H-Glc-Rha-CH_3_]^−^ and m/z 271.0605 [M-H-Glc-Rha-OCH_3_]^−^. According to the literature review and fragment information, it is inferred that the compound is Hesperidin, and the cleavage process is shown in **Figure 3A**.

#### 3.3.2 Esters

Ester compounds refer to a class of organic compounds formed by the reaction of acids and alcohols, and the most important feature is the existence of ester bonds. Under mass spectrometry conditions, the most characteristic fragmentation mode is also the cleavage of the ester bond, accompanied by the cleavage of the alkyl group.

A total of 23 ester compounds were characterized in the XHP, of which 8 belong to *P. ginseng*, 7 belong to *B. chinense*, 6 belong to *S. baicalensis*, 6 belong to *E. sinica*, 4 belong to *C. cassia*, and 1 each of the chemical components belonging to *L. chinense*, *R. glutinosa*, *S. glabra*, and *A. macrocephala*. Taking compound 19 as an example, in negative ion mode, its quasi-molecular ion peak is m/z 161.0451 [M-H]^−^, and the elemental composition of this compound is C_6_H_10_O_5_. The parent ion removed one molecule of COOCH_3_ to obtain m/z 103.0401 [M-COOCH_3_]^−^, one molecule of hydroxyl to obtain m/z 145.0506 [M-OH]^−^, one molecule of methoxy to obtain m/z 131.0350 [M-OCH_3_]^−^, and simultaneously removed one molecule of hydroxyl and one molecule of methoxy to obtain m/z 115.0401 [M-OH-OCH_3_]^−^. According to the literature review and fragment information, it is inferred that the compound is dimethyl L-malate, and the cleavage process is shown in **Figure 3B**.

#### 3.3.3 Organic acids

Organic acid compounds widely exist in plants and are commonly known to mediate strong pharmacological antioxidant, antibacterial, and anti-inflammatory effects. They are easy to lose CO, CO_2_, −COOH, H_2_O, etc. in mass spectrometry, resulting in fragments.

A total of 34 organic acid compounds were characterized in XHP, among which 9 belong to *C. cassia*, 7 belong to *B. chinense*, 7 belong to *E. sinica*, 6 belong to *A. macrocephala*, 6 belong to *L. chinense*, 4 belong to *R. glutinosa*, 4 belong to *G. uralensis*, 4 belong to *P. ginseng*, 4 belong to *S. glabra*, and 3 chemical components belonging to *S. baicalensis*. Taking compound 11 as an example, in the negative ion mode, its quasi-molecular ion peak is m/z 133.0141 [M-H]^−^, and the elemental composition of this compound is C_4_H_6_O_5_. The parent ion removes one molecule of carboxyl to obtain m/z 89.0244 [M-COOH]^−^, or the parent ion removes three hydroxyl groups accordingly to obtain m/z 117.0193 [M-OH]^−^, m/z 101.0244 [M-2OH]^−^, and m/z 85.0295 [M-3OH]^−^. According to the literature review and fragment information, it is inferred that the compound is (2S)-2-hydroxybutanedioic acid, and the cracking process is shown in **Figure 3C**.

#### 3.3.4 Saponins

Saponins are a class of glycosides that have valuable biological activities including antibacterial, antipyretic, sedative, and anticancer effects. The main method of saponin mass spectrometry fragmentation is the loss of glycosyl group, which is also accompanied by the loss of polar functional groups such as hydroxyl, methyl, and methoxy groups in the aglycone part.

A total of 16 saponins were identified in XHP, including 6 chemical components belonging to *P. ginseng*, 5 chemical components belonging to *B. chinense*, and 5 chemical components belonging to *G. uralensis*. Taking compound 118 as an example, in the negative ion mode, its quasi-molecular ion peak is m/z 927.5380 [M-H]^−^, and the elemental composition of this compound is C_49_H_81_O_19_. The parent ion removes one molecule of rhamnose and one molecule of glucose, respectively, thus obtaining m/z 781.4734 [M-H-Rha]^−^ and m/z 765.4788 [M-H-Glc]^−^, then removes one molecule of glucose respectively and a molecule of rhamnose, getting m/z 619.4204 [M-H-Glc-Rha]^−^, and continues to remove a molecule of glucose, getting m/z 457.3670 [M-H-Rha-2Glc]^−^. According to the literature review and fragment information, it is inferred that the compound is Saikosaponin F, and the cracking process is shown in **Figure 3D**.

#### 3.3.5 Aldehydes

Compounds in which carbonyl carbon is connected to hydrogen and hydrocarbon groups are called aldehydes, which can be further divided into aliphatic aldehydes, ester cyclic aldehydes, aromatic aldehydes, and terpene aldehydes. Under mass spectrometry conditions, aldehydes often have the characteristic fragmentation mode of the hydrocarbon moiety as the main fragmentation mode.

A total of nine aldehyde compounds were identified in XHP, including five compounds belonging to *B. chinense*, three compounds belonging to *C. cassia*, three compounds belonging to *E. sinica*, one compound belonging to *R. glutinosa*, and one compound belonging to *S. baicalensis*. Taking compound 162 as an example, in positive ion mode, its quasi-molecular ion peak is 123.0437 [M+H]^+^, and the elemental composition of this compound is C_7_H_6_O_2_. The parent ion first removes one molecule of aldehyde group to obtain m/z 93.0335 [M-CHO]^+^ and then removes one molecule of hydroxyl group to obtain m/z 77.0386 [M-CHO-OH]^+^. According to the literature review and fragment information, it is inferred that the compound is salicylaldehyde, and the cracking process is shown in **Figure 3E**.

#### 3.3.6 Coumarins

Coumarins are mainly divided into simple coumarins, furanocoumarins, and pyranocoumarins. Substituents mainly include hydroxyl, methoxy, hydroxyisopropyl, isopentenyl, modified isopentenyl, and ethers formed by the latter two and coumarin. The main cracking method is to first lose the unique substituent according to the different substituents and then continuously lose two molecules of CO on the pyran ring main.

A total of five coumarin compounds were identified in XHP, of which five belong to *B. chinense*, one belongs to *A. macrocephala*, one belongs to *G. uralensis* and *L. chinense*, one belongs to *G. uralensis*, and one chemical composition belongs to *E. sinica*. Taking compound 37 as an example, in negative ion mode, its quasi-molecular ion peak is 207.0291 [M-H]^−^, and the elemental composition of this compound is C_10_H_8_O_5_. The parent ion accordingly removes one molecule of methoxy group m/z 177.0185 [M-OCH_3_]^−^ and one molecule of C_2_H_2_CO m/z 153.0184 [M-C_2_H_2_CO]^−^ to obtain m/z 123.0079 [M-OCH_3_-C_2_H_2_CO]^−^. According to the literature review and fragmentation information, it is inferred that the compound is Fraxetin, and the cracking process is shown in **Figure 3F**.

#### 3.3.7 Alkaloids

Alkaloids are a class of nitrogen-containing basic organic compounds that exist in nature, and their molecular ion peaks often exist in the form of addition of [M+H]^+^. Under mass spectrometry conditions, NH_3_, CH_3_, CO, and CH_4_O produce corresponding characteristic neutral fragments.

A total of five alkaloids were identified in XHP, of which three belongs to *E. sinica*, one belongs to *L. chinense*, and one belongs to *L. chinense*. Taking compound 31 as an example, in positive ion mode, its quasi-molecular ion peak is 166.1225 [M+H]^+^, and the elemental composition of this compound is C_10_H_15_NO. The parent ion first removes a molecule of hydroxyl to obtain m/z 150.1277 [M-OH]^+^, then removes a molecule of methyl to obtain m/z 136.1121 [M-OH-CH_3_]^+^, and finally removes a molecule of amino group to obtain m/z 119.0855 [M-OH-CH_3_-NH_2_]^+^. According to the literature review and fragment information, it is inferred that the compound is Pseudoephedrine, and the cracking process is shown in **Figure 3G**.

### 3.4 Network pharmacology analysis

#### 3.4.1 Construction of the herb–component–target network

In the study, 1,158 targets related to XHP and 8,841 targets about ALD were acquired, of which 457 targets were the XHP-ALD overlapped targets, including 284 targets from *B. chinense*, 235 targets from *S. baicalensis*, 340 targets from *E. sinica*, 156 targets from *C. cassia*, 215 targets from *R. glutinosa*, 105 targets from *S. glabra*, 95 targets from *A. macrocephala*, 212 targets from *L. chinense*, 251 targets from *P. ginseng*, and 299 targets from *G. uralensis*.

**Figure 4:**
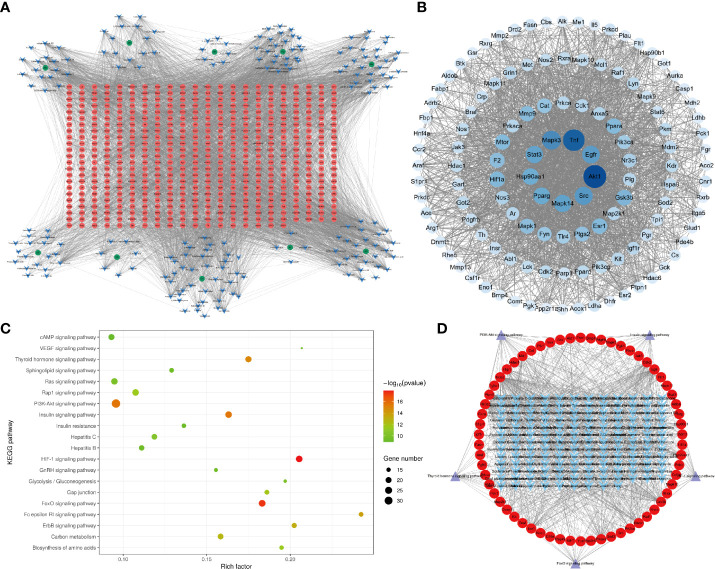
Network pharmacology of XHP. **(A)** The herb–component–target network of XHP in the treatment of ALD. Green represents the herb, blue represents the active components, and red represents the targets. **(B)** PPI network of XHP in the treatment of ALD. Network nodes represent different proteins. Edges represent protein–protein associations. **(C)** Kyoto Encyclopedia of Genes and Genomes (KEGG) pathway enrichment analysis of XHP in the treatment of ALD. **(D)** The component–target–pathway network of XHP in the treatment of ALD. Blue represents the active component, red represents the targets, and purple represents the pathway.

The herb–component–target network was constructed by Cytoscape 3.8.2 software, as presented in **Figure 4A**. This analysis demonstrates the XHP network that includes 704 nodes and 5,367 edges, with an average component degree of 27.89. There were 85 components with higher than average degree value, which indicated that these components potentially regulate multiple targets to mediate several therapeutic effects. These targets were selected for PPI network analysis to further elucidate this complex.

#### 3.4.2 PPI network analysis

The PPI network consisted of 123 nodes and 2,258 edges (**Figure 4B**). The first circle from the inside out was targets with degree >100, the second circle was targets with degree >56, the third circle was targets with degree >42, and the rest were in the last circle. The larger the node and the darker the color, the higher likelihood the node has; that is, the node is more likely to be a key target for XHP to prevent ALD. Targets with a degree greater than the average were imported into the Omicsbean database for KEGG enrichment analysis.

#### 3.4.3 KEGG metabolic pathway enrichment analysis

KEGG metabolic pathway enrichment analysis revealed that a total of 125 related metabolic pathways were significantly (*p* < 0.05) associated with XHP treatment. These pathways were sorted according to the p-value from small to large and used to obtain KEGG-enriched pathway map, as shown in **Figure 4C**. Highly enriched pathways associated with the effects of XHP as an ALD treatment included the HIF-1 (24), FoxO, PI3K-Akt (24, 25), insulin, and thyroid hormone signaling pathways (26). These results are consistent with previous observations, as alcohol has been shown to inhibit insulin signaling pathway (27), leading to glucose metabolism disorders. Additionally, inflammation is a key factor in the development of insulin resistance. It is widely understood that alcohol and its metabolites release a large number of inflammatory factors into the human body, which provides further support that the insulin pathway is involved in mediating the protective effects of XHP. The other four signaling pathways were mainly involved in regulating lipid metabolism.

#### 3.4.4 Construction of the component–target–pathway network

The component–target–pathway network diagram was constructed by Cytoscape 3.8.2. based on the results of the KEGG enrichment analysis. **Figure 4D** shows that there were 153 components with an average degree value of 5.6 and 75 components with an above average degree value. There were 62 target nodes, and the average degree value was 9.25. There were 29 targets with a degree higher than average.

#### 3.4.5 Molecular docking

**Figure 5:**
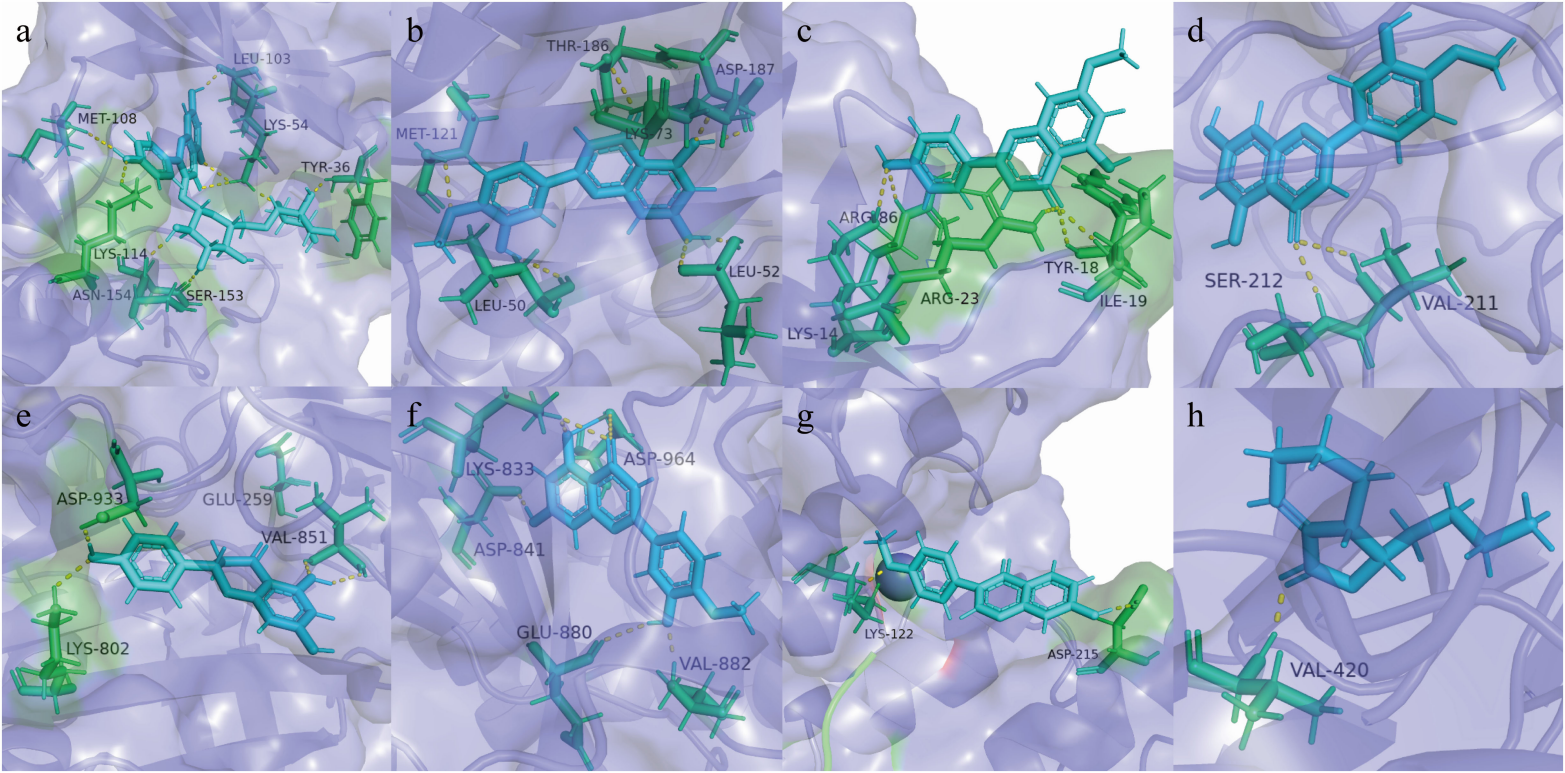
Molecular models of core targets binding to the related compound. **(A)** Mapk1 and Nicotiflorin, **(B)** Mapk3 and Diosmetin, **(C)** Akt1 and Genkwanin, **(D)** Map2k1 and Diosmetin, **(E)** Pik3ca and Naringenin, **(F)** Pik3cg and Diosmetin, **(G)** Raf1 and Formononetin, and **(H)** Prkca and Neocnidilide.

Combining the results of PPI and KEGG analysis, the targets with high PPI network moderate value and high rich factor in KEGG analysis were selected for molecular docking with their related chemical components. These eight targets were Mapk1 (PDB ID: 1pme), Mapk3 (PDB ID: 3fhr), Akt1 (PDB ID: 1h10), Map2k1 (PDB ID: 1s9j), Pik3ca (PDB ID: 4jps), Pik3cg (PDB ID: 1e8z), Raf1 (PDB ID: 3iqv), and Prkca (PDB ID: 4ra4). The docking results between eight targets and related compounds are shown in **Supplementary Table S2**, and the best combination result of each target is shown in **Figure 5**. The higher is the docking score, the better the combination of the compound and the target is predicted to be (28). The compounds with the highest docking score with these eight targets included Nicotiflorin, Diosmetin, Genkwanin, Naringenin, Formononetin, and Neocnidilide, five of which were flavonoids. Previous reports have suggested that the strong antioxidant activity of flavonoids makes these compounds increasingly attractive as potential therapeutic options for ALD patients (29, 30). The mechanism of flavonoids for liver protection involves anti-oxidative stress, regulation of liver lipid metabolism, and inhibition of liver inflammation, which are consistent with the results of our pharmacodynamic experiments.

#### 3.4.6 Results of proteomics

**Figure 6:**
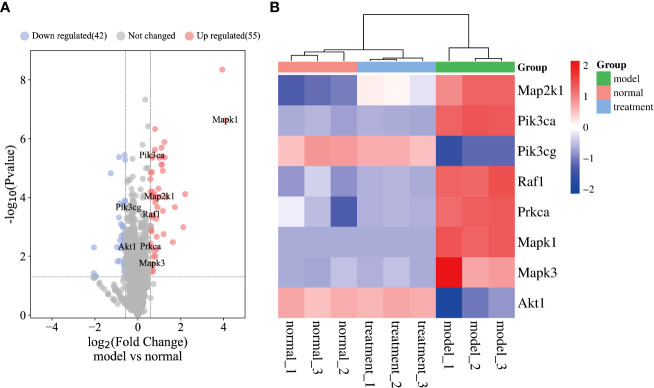
Results of proteomics. **(A)** The volcano plot of model group vs control group. **(B)** The heatmap of key targets.

The proteomic analysis results are visualized in in **Figure 6**. A total of 2,657 protein groups were identified in all three experimental groups (normal, model, and treatment). Compared with the normal group, 97 proteins in the model group were significantly different, 55 of which were upregulated and 42 proteins were downregulated (**Figure 6A**). Additionally, eight key targets used in the molecular docking experiments were also identified as significantly differentially expressed proteins. Among them, Akt1 and Pik3cg were downregulated, while the other six targets were upregulated. **Figure 6B** demonstrates the significant differences in protein expression between all three experimental groups. In addition, the protein expression levels of eight key targets in the treatment group showed a back regulation. Mapk1, Mapk3, and Map2k1 belong to the main members of the MAPK family (31), which play an important role in many pathological processes such as cell growth, differentiation, and the inflammatory response. Taken together, inhibiting protein expression of MAPK has the potential to effectively alleviate inflammation alcoholic liver disease. Akt1 regulates cell proliferation and growth, reduces the expression of Akt1 protein in the body, and effectively inhibits the proliferation of liver cancer cells and promotes their apoptosis (32). The high expression of Pik3ca and Prkca could cause liver cancer cells to proliferate. The function of Pik3cg gene was related to cell growth, survival, proliferation, movement, and morphology. Lipid accumulation in the liver impairs Raf1 and causes insulin resistance.

## 4 Conclusion

In this study, XHP treatment significantly improved the alcohol-induced liver injury in mice. This study also identified 199 compounds that make up XHP utilizing UPLC-QTOF/MS, including 43 flavonoids, 34 organic acids, 25 esters, 16 saponins, 16 glycosides, 11 ketones, 9 aldehydes, 5 coumarins, and 5 alkaloids. Additionally, the hepatoprotective mechanism of XHP was further investigated by network pharmacology. Among them, 103 targets regulated by 163 chemical XHP components may play an important role in the liver protection effect of XHP. The core targets and their related compounds including Nicotiflorin, Diosmetin, Genkwanin, Naringenin, Formononetin, and Neocnidilide were verified by molecular docking experiments. The eight key targets were further verified by proteomics, which provided a mechanistic insight into the effects of XHP as an ALD treatment. In conclusion, this study demonstrated that XHP exerted protective effects in an animal model of ALD and enriched our knowledge of the chemical constituents and hepatoprotective mechanism of XHP. However, the active components, active targets, and signaling pathways predicted in this study should be further confirmed and validated in future studies.

## Data availability statement

The original contributions presented in the study are included in the article/**Supplementary Material**. Further inquiries can be directed to the corresponding authors.

## Ethics statement

The animal study was reviewed and approved by Nanjing Agricultural University.

## Author contributions

XC, MD, and KW, data curation and writing-original draft preparation. CD and YL, methodology and conceptualization. RY, BZ, and ZL, data validation and curation. YY and XY, writing–reviewing, visualization, and editing. YL, WL, and YW, conceptualization, funding acquisition, and project administration. All authors contributed to the article and approved the submitted version.
