# Supplementary material for: Study of Xuanhuang Pill in protecting against alcohol liver disease using ultra-performance liquid chromatography/time-of-flight mass spectrometry and network pharmacology
Source: Front Endocrinol (Lausanne). 2023 Apr 4;14:1175985. doi: 10.3389/fendo.2023.1175985 (PMC10111029; doi:10.3389/fendo.2023.1175985)
Supplement: Supplementary file 1 [file DataSheet_1.docx]

Supplementary Material

Study of Xuanhuang Pill in protecting against alcohol liver disease by ultra-performance liquid chromatography/ time of flight mass spectrometry and network pharmacology

Xuejie Cui*, Chen Dai, Rachel Y. H. Yang, Yi Wu, Maobo Du, Yuhong Liu

*** Correspondence:** Xuejie Cui: c15562588660@163.com

# Supplementary Table 1. UPLC-Q-TOF-MS identification of XHP

| No. | t_R_/min | Predicted formula | Neutral  mass | Observed  m/z | mass error  （mDa） | MS/MS | Adduct | Component name | Herb | Structure |
| --- | --- | --- | --- | --- | --- | --- | --- | --- | --- | --- |
| 1 | 0.88 | C_6_H_12_O_6_ | 180.0634 | 203.0522 | -0.4 | [M-4OH] 139.0725[C_6_H_12_O_2_Na]^+^, [M-OH] 187.0573[C_6_H_12_O_5_Na]^+^, [M-OH-CH_2_OH] 134.0579[C_5_H_10_O_4_]^+^_,_ [M-2OH] 148.0736[C_6_H_12_O_4_]^+^ | +Na | Methose | BZ | glycosides |
| 2 | 0.88 | C_6_H_14_O_6_ | 182.0790 | 181.0714 | -0.4 | [M-OH] 163.0602[C_6_H_11_O_5_]^-^ | -H, +HCOO | Mannitol | DH | others |
| 3 | 0.92 | C_5_H_11_NO_2_ | 117.0790 | 118.0860 | -0.2 | [M-OH] 100.0757[C_5_H_10_NO]^+^, [M-NH_2_-CH_3_] 85.0284[C_4_H_5_O_2_]^+^, [M-CH_3_] 102.0550[C_4_H_8_NO_2_]^+^, [M-OH-CH_3_] 86.0600[C_4_H_8_NO]^+^ | +H, +Na | (2S)-2-Azaniumyl-3-methylbutanoate | BZ | organic acids |
| 4 | 0.92 | C6H12O6 | 180.0634 | 179.0560 | -0.1 | [M-OH] 161.0451[C_6_H_9_O_5_]^-^, [M-2OH-OCH_3_] 113.0243[C_5_H_5_O_3_]^-^, [M-C_2_H_6_O_3_] 101.0241[C_4_H_5_O_3_]^-^, [M-2OH] 143.0347[C_6_H_7_O_4_]^-^ | -H | beta-D-Galactose | CH | glycosides |
| 5 | 0.93 | C_24_H_42_O_21_ | 666.2219 | 665.2152 | 0.6 | [M-C_6_H_10_O_5_] 503.1621[C_18_H_31_O_16_]^-^, [M-C_12_H_20_O_10_] 341.1092[C_12_H_21_O_11_]^-^, [M-C_14_H_26_O_11_] 295.06763[C_10_H_15_O_10_]^-^, [M-C_18_H_30_O_15_] 179.0557[C_6_H_11_O_6_]^-^ | -H | Stachyose | DH | glycosides |
| 6 | 0.95 | C_6_H_12_O_5_ | 164.0685 | 209.0660 | -0.6 | [M-2OH-CH_3_] 113.0243[C_5_H_5_O_3_]^-^, [M-OH-CHOHCH_3_] 101.0241[C_4_H_5_O_3_]^-^, [M-CHOHCH_3_] 119.0347[C_4_H_7_O_4_]^-^, [M-OH-CH_3_] 129.0189[C_5_H_5_O_4_]^-^ | +HCOO | L-Rhamnose | CH | glycosides |
| 7 | 0.95 | C_36_H_48_O_20_ | 800.2739 | 845.2732 | 1.1 | [M-C_18_H_31_O_11_] 377.0863[C_18_H_17_O_9_]^-^, [M-C_24_H_27_O_9_] 341.1093[C_12_H_21_O_11_]^-^, [M-C_12_H_21_O_6_] 539.1390[C_24_H_27_O_14_]^-^, [M-C_29_H_37_O_14_] 191.0560[C_7­_H_11_O_6_]^-^ | +HCOO | Cistanoside A | DH | glycosides |
| 8 | 0.95 | CH_2_Cl_2_ | 83.9534 | 84.9596 | -1.0 | [M+H] 84.9842[CH_3_Cl_2_]^+^ | +H | Dichloromethane | MH | others |
| 9 | 0.97 | C_5_H_9_NO_2_ | 115.0633 | 116.0701 | -0.5 | [M-OH] 98.0600[C_5_H_8_NO]^+^, [M-CH_2_-NH] 85.0284[C_4_H_5_O_2_]^+^, [M-OH-NH] 83.0491[C_5_H_7_O]^+^, [M-C_2_H_4_] 88.0393[C_3_H_6_NO_2_]^+^ | +H | Prolinum | BZ | organic acids |
| 10 | 1.04 | C_5_H_7_NO_3_ | 129.0426 | 128.0349 | -0.4 | [M-OH] 110.0245[C_5_H_4_NO_2_]^-^, [M-CONH] 85.0291[C_4_H_5_O_2_]^-^ | -H | L-Pyroglutamic acid | DH | organic acids |
| 11 | 1.21 | C_4_H_6_O_5_ | 134.0215 | 133.0141 | -0.1 | [M-2OH] 101.0244[C_4_H_5_O_3_]^-^, [M-COOH] 89.0244[C_3_H_5_O_3_]^-^, [M-OH] 117.0193[C_4_H_5_O_4_]^-^, [M-3OH] 85.0295[C_4_H_5_O_2_]^-^ | -H | (2S)-2-Hydroxybutanedioic acid | GQ | organic acids |
| 12 | 1.30 | C_18_H_32_O_16_ | 504.1690 | 549.1682 | 1.0 | [M-C_6_H_11_O_5_] 341.1093[C_12_H_21_O_11_]^-^, [M-C_12_H_21_O_10_] 179.0558[C_6_H_11_O_6_]^-^, [M-C_6_H_15_O_5_] 337.0778[C_12_H_17_O_11_]^-^, [M-OH] 485.1512[C_18_H_29_O_15_]^-^ | +HCOO | Raffinose | DH | glycosides |
| 13 | 1.33 | C_5_H_4_O_2_ | 96.0211 | 97.0280 | -0.4 | [M+H] 97.0280[C_5_H_5_O_2_]^+^ | +H | 3-Furaldehyde | CH | aldehydes |
| 14 | 1.40 | C_4_H_6_O_2_ | 86.0368 | 85.0294 | -0.1 | [M-H] 85.0294[C_4_H_5_O_2_]^-^ | -H | gamma-Butyrolactone | MH | esters |
| 15 | 1.65 | C_9_H_12_N_2_O_6_ | 244.0695 | 243.0620 | -0.3 | [M-NH-OH] 212.0562[C_9_H_10_NO­_5_]^-^, [M-OH-C_4_H_5_N_2_O_3_] 113.0240[C_5_H_5_O_3_]^-^, [M-C_2_H_2_NO] 188.0560[C_7_H_10_NO_5_]^-^, [M-2OH] 211.0710[C_9_H_11_N_2_O_4_]^-^ | -H | Uridine | DH | glycosides |
| 16 | 1.78 | C_6_H_6_O_4_ | 142.0266 | 187.0247 | -0.2 | [M-CH_2_OH] 111.0087[C_5_H_3_O_3_]^-^, [M-OH] 125.0242[C_6_H_5_O_3_]^-^ | +HCOO | Sumiki's acid | DH | organic acids |
| 17 | 1.79 | C_6_H_8_O_4_ | 144.0423 | 143.0345 | -0.5 | [M-OCH_2_] 111.0087[C_5_H_3_O_3_]^-^, [M-OH] 125.0242[C_6_H_5_O_3_]^-^ | -H | 2,6,10-trimethyl-dodecane | GC, MH | others |
| 18 | 1.88 | C_6_H_4_O_4_ | 140.0110 | 185.0084 | -0.8 | [M-CHO] 113.0240[C_5_H_5_O_3_]^-^, [M-COO] 97.0290[C_5_H_5_O_2_]^-^, [M-O-OH] 109.0286[C_6_H_5_O_2_]^-^ | +HCOO | Cumalic acid | GQ | organic acids |
| 19 | 1.94 | C_6_H_10_O_5_ | 162.0528 | 161.0451 | -0.5 | [M-OH-OCH_3_] 115.0401[C_5_H_7_O_3_]^-^, [M-COOCH_3_] 103.0401[C_4_H_7_O_3_]^-^, [M-OH] 145.0506[C_6_H_9_O_4_]^-^, [M-OCH_3_] 131.0350[C_5_H_7_O_4_]^-^ | -H | Dimethyl L-malate | FL | esters |
| 20 | 2.16 | C_6_H_13_NO_2_ | 131.0946 | 130.0873 | 0.0 | [M-NH] 115.0763[C_6_H_11_O_2_]^-^ | -H | l-Isoleucine | BZ | organic acids |
| 21 | 2.62 | C_5_H_6_O_2_ | 98.0368 | 99.0436 | -0.5 | [M+H] 99.0436[C_5_H_7_O_2_]^+^ | +H | Furfuryl alcohol | CH | others |
| 22 | 3.15 | C_9_H_11_NO_2_ | 165.0790 | 164.0712 | -0.5 | [M-NH_2_] 149.0605[C_9_H_9_O_2_]^-^, [M-NH_2_-COOH] 103.0548[C_8_H_7_]^-^ | -H | L-(-)-Phenylalanine | BZ | organic acids |
| 23 | 3.21 | C_14_H_18_O_7_ | 298.1052 | 343.1029 | -0.6 | [M-C_6_H_11_O_5_] 135.0445[C_8_H_7_O_2_]^-^, [M-C_5_H_9_O_3_] 181.0502[C_9_H_9_O_4_]^-^, [M-C_7_H­_11_O_5_] 123.0446[C_7_H_7_O_2_]^-^, [M-C_6_H_11_O_4_] 151.0394[C_8_H_7_O_3_]^-^ | +HCOO | Picein | MH | glycosides |
| 24 | 3.40 | C_7_H_6_O_4_ | 154.0266 | 153.0190 | -0.4 | [M-COOH] 109.0287[C_6_H_5_O_2_]^-^, [M-OH] 137.0241[C_7_H_5_O_3_]^-^, [M-COOH-OH] 91.0182[C_6_H_3_O]^-^ | -H | Protocatechuic acid | MH, GC  GZ | organic acids |
| 25 | 3.41 | C_6_H_6_O_2_ | 110.0368 | 109.0291 | -0.4 | [M-H] 109.0291[C_6_H_5_O_2_]^-^ | -H | 5-Methylfurfural | CH | aldehydes |
| 26 | 3.43 | C_10_H_13_N_5_O_4_ | 267.0968 | 290.0877 | 1.8 | [M-NH_2_] 252.0850[C_10_H_12_N_4_O_4_]^+^, [M-NH_2_-2OH-CH_2_OH] 188.0693[C_9_H_8_N_4_O]^+^, [M-NH_2_-C_2_H_3_O_2_] 192.0642[C_8_H_8_N_4_O_2_]^+^, [M-NH_2_-CHO_2_] 206.0798[C_9_H_10_N_4_O_2_]^+^ | +Na | L-Adenosine | RS | glycosides |
| 27 | 3.44 | C_8_H_8_O_4_ | 168.0423 | 167.0348 | -0.2 | [M-COOH] 123.0446[C_7_H_7_O_2_]^-^, [M-CH_3_] 153.0186[C_7_H_5_O_4_]^-^ | -H | Vanillic acid | CH | organic acids |
| 28 | 3.68 | C_9_H_7_NO_2_ | 161.0479 | 206.0452 | -0.7 | [M-OH] 144.0446[C_9_H_6_NO]^-^, [M-COOH] 116.0497[C_8_H_6_N]^-^ | +HCOO | Indole-3-carboxylic acid | GC | organic acids |
| 29 | 3.79 | C_6_H_6_O_3_ | 126.0317 | 127.0398 | 0.0 | [M-OH] 109.0284[C_6_H_5_O_2_]^+^, [M-CHO] 99.04406[C_5_H_7_O_2_]^+^ | +H | 5-Hydroxymethylfurfural | DH, MH | aldehydes |
| 30 | 3.80 | C_20_H_23_N_7_O_7_ | 473.1659 | 474.1739 | 0.8 | [M-C_10_H_12_N_6_O_6_] 162.0913[C_10_H_12_NO]^+^, [M-C_15_H_9_NO_4_] 327.1200[C_15_H_15_N_6_O_3_]^+^, [M-C_11_H_14_N_6_O_7_] 132.0808[C_9_H_10_N]^+^, [M-C_7_H_8_N_3_O_5_] 260.1268[C_13_H_16_N_4_O_2_]^+^ | +H | Folinic acid | RS | organic acids |
| 31 | 3.97 | C_10_H_15_NO | 165.1154 | 166.1225 | -0.1 | [M-OH] 150.1277[C_10_H_16_N]^+^, [M-OH-CH_3_] 136.1121[C_10_H_16_N]^+^, [M-OH-CH_3_-NH_2_] 119.0855[C_9_H_11_]^+^ | +H | Pseudoephedrine | MH | alkaloids |
| 32 | 3.99 | C_10_H_15_NO | 165.1154 | 166.1227 | 0.0 | [M-C_2_H_8_O] 118.0651[C_8_H_8_N]^+^, [M-C_3_H_11_NO] 89.0386[C_7_H_5_]^+^, [M-C_3_H_10_NO] 90.0464[C_7_H_6_]^+^, [M-C_4_H_11_NO] 77.0386[C_6_H_5_]^+^ | +H | Eciphin | MH | alkaloids |
| 33 | 4.10 | C_21_H_28_O_13_ | 488.1530 | 487.1449 | -0.8 | [M-OH] 472.1567[C_21_H_28_O_12_]^-^, [M-C_15_H_20_O_5_] 191.0193[C_6_H_7_O_7_]^-^, [M-C_9_H_16_O_8_] 235.0624[C_12_H_11_O_5_]^-^, [M-C_13_H_15_O_7_] 204.0642[C_8_H_12_O_6_]^-^ | -H | Cistanoside F | DH | glycosides |
| 34 | 4.17 | C_6_H_12_O_2_ | 116.0837 | 115.0762 | -0.3 | [M-OH] 97.0653[C_6_H_9_O]^-^, [M-C_2_H_5_] 85.0290[C_4_H_5_O_2_]^-^, [M-OH-CH_3_] 85.0654[C_5_H_9_O]^-^ | -H, +HCOO | hexanoic acid | CH, GZ  HQ, MH | organic acids |
| 35 | 4.17 | C_5_H_8_O | 84.0575 | 129.0551 | -0.6 | [M+HCOO] 129.0551[C_6_H_9_O_3_]^-^ | +HCOO | Tiglaldehyde | MH | aldehydes |
| 36 | 4.21 | C_11_H_17_NO | 179.1310 | 180.1381 | -0.2 | [M-OH-CH_3_] 146.0964[C_10_H_12_N]^+^, [M-C_4_H_11_NO] 91.0536 [C_7_H_7_]^+^, [M-OH-2CH_3_] 132.0808[C_9_H_10_N]^+^, [M-C_4_H_13_NO] 89.0386[C_7_H_5_]^+^ | +H | Methylephedrine | MH | alkaloids |
| 37 | 4.23 | C_10_H_8_O_5_ | 208.0372 | 207.0291 | -0.8 | [M-OCH_3_-1,4A] 123.0079 [C_6_H_3_O_3_]^-^, [M-1,4] 153.0184[C_7_H_5_O_4_]^-^, [M-OCH_3_] 177.0185[C_9_H_5_O_4_]^-^ | -H | Fraxetin | CH | coumarins |
| 38 | 4.26 | C_28_H_32_O_16_ | 624.1690 | 623.1625 | 0.7 | [M-glc-rha-1,3] 165.0551[C_9_H_9_O_3_]^-^, [M-rha-CH_3_-1,3A] 314.1014[C_14_H_18_O_8_]^-^,[M-glc-rha-1,3b] 151.0027[C_7_H_3_O_4_]^-^ | -H | Narcissoside | CH, GC | flavonoids |
| 39 | 4.32 | C_6_H_6_O | 94.0419 | 93.0341 | -0.5 | [M-H] 93.0341[C_6_H_5_O]^-^ | -H | Hydron | CH, GZ | others |
| 40 | 4.42 | C_21_H_20_O_10_ | 432.1057 | 433.1129 | 0.0 | [M-glc-1,4A-OH] 131.0488[C_9_H_4_O]^+^, [M-0,4A-glc] 163.0376[C_9_H_7_O_3_]^+^ | +H | Vitexin | GC, MH | flavonoids |
| 41 | 4.56 | C_8_H_12_N_2_ | 136.1001 | 137.1070 | -0.3 | [M+H] 137.1070[C_8_H_13_N_2_]^+^ | +H | Tetramethylpyrazine | HQ, MH | others |
| 42 | 4.74 | C_9_H_11_NO_3_ | 181.0838 | 182.0811 | -0.1 | [M-OH-NH_2_] 147.0441[C_9_H_7_O_2_]^+^, [M-COOH] 136.0757[C_8_H_10_NO]^+^, [M-COOH-NH_2_] 119.0491[C_8_H_7_O]^+^, [M-OH-NH_2_-COOH] 89.0386[C_7_H_5_]^+^ | +H | L- (-)-Tyrosine | BZ | organic acids |
| 43 | 4.84 | C_9_H_8_O_4_ | 180.0423 | 179.0347 | -0.3 | [M-COOH] 135.0443[C_8_H_­7_O_2_]^-^, [M-OH] 161.0239[C_9_H_5_O_3_]^-^, [M-C_2_H_2_COOH] 109.0287[C_6_H_5_O_2_]^-^ | -H | Caffeic acid | CH | organic acids |
| 44 | 4.93 | C_21_H_20_O_9_ | 416.1107 | 415.1038 | 0.3 | [M-glc] 253.0506[C_15_H_9_O_4_]^-^, [M-glc-1,3A] 117.0339[C_8_H_5_O]^-^ | -H | Puerarin | CH | flavonoids |
| 45 | 4.93 | C_17_H_16_O_4_ | 284.1049 | 307.0955 | 1.5 | [M-CH_3_-OCH_3_] 240.0786[C_15_H_12_O_3_]^+^, [M-CH_3_-OH] 254.0943[C_16_H_14_O_3_]^+^, [M-CH_3_] 270.0892[C_16_H_14_O_4_]^+^, [M-C_6_H_4_OCH_3_-CH_3_] 164.0473[C_9_H_8_O_3_]^+^ | +Na | Glypallichalcone | GC | ketones |
| 46 | 4.97 | C_10_H_10_O_4_ | 194.0579 | 239.0563 | 0.2 | [M-CHCOOH] 135.0445[C_8_H_7_O_2_]^-^, [M-COOH] 149.0600[C_9_H_9_O_2_]^-^, [M-OH] 177.0552[C_10_H_9_O_3_]^-^, [M-CHCOOH] 134.0366[C_8_H_6_O_2_]^-^ | +HCOO | Ferulic acid | GZ | organic acids |
| 47 | 5.05 | C_6_H_6_ | 78.0470 | 123.0447 | -0.4 | [M+HCOO] 123.0447[C_7_H_7_O_2_]^-^ | +HCOO | Benzene | MH | others |
| 48 | 5.06 | C_27_H_30_O_15_ | 594.1585 | 593.1524 | 1.2 | [M-glc-rha-1,3B] 151.0030 [C_7_H_3_O_4_]^-^ | -H | Nicotiflorin | GC | flavonoids |
| 49 | 5.09 | C_5_H_5_N | 79.0422 | 124.0402 | -0.2 | [M+HCOO] 124.0402[C_6_H_6_NO_2_]^-^ | +HCOO | Py | CH | others |
| 50 | 5.14 | C_22_H_22_O_10_ | 446.1213 | 445.1148 | 0.8 | [M-glc-OCH_3_] 253.0505 [C_15_H_9_O_4_]^-^, [M-glc] 281.0454[C_16_H_9_O_5_]^-^, [M-CH_3_] 430.0909[C_21_H_18_O_10_]^-^, [M-glc-1,3B] 151.0032 [C_7_H_3_O_4_]^-^, [M-glc-CH_3_-1,3A] 119.0495[C_8_H_7_O]^-^ | -H | Tilianine | MH | flavonoids |
| 51 | 5.43 | C_35_H_46_O_20_ | 786.2584 | 785.2517 | 0.8 | [M-C_9_H_6_O_3_] 623.2191[C_26_H_39_O_17_]^-^, [M-C_14_H_6_O_3_] 433.11406[C_21_H_21_O_10_]^-^, [M-C_20_H_34_O_15_] 271.0610[C_15_H_11_O_5_]^-^, [M-C_26_H_40_O_17_] 161.0244[C_9_H_5_O_3_]^-^ | -H | Echinacoside | DH | glycosides |
| 52 | 5.44 | C_29_H_36_O_15_ | 624.1690 | 625.2134 | 0.7 | [M-C_14_H_20_O_7_] 325.0918[C_15_H_17_O_8_]^+^, [M-C_20_H_30_O_12_] 163.0390[C_9_H_7_O_3_]^+^, [M-C_8_H_10_O_3_] 471.1497[C_21_H_27_O_12_]^+^, [M-C_6_H_10_O_4_] 479.1548[C_23_H_27_O_11_]^+^ | +H | Acteoside | DH | glycosides |
| 53 | 5.52 | C_8_H_8_O_2_ | 136.0524 | 135.0447 | -0.4 | [M-CH_3_] 121.0289[C_7_H_5_O]^-^, [M-COCH_3_] 93.0341[C_6_H_5_O]^-^, [M-OH] 117.0339[C_8_H_5_O]^-^, [M-OH-CH_3_] 105.0337[C_7_H_5_O]^-^ | -H | Piceol | MH | ketones |
| 54 | 5.76 | C_8_H_8_O | 120.0575 | 119.0497 | -0.5 | [M-H] 119.0497[C_8_H_7_O]^-^ | -H | Hyacinthin | CH, GZ  HQ | aldehydes |
| 55 | 5.77 | C_9_H_6_O_2_ | 146.0368 | 147.0440 | -0.1 | [M-1,4]95.0485[C_6_H_7_O]^-^ | +H | Coumarin | GZ, CH  MH | coumarins |
| 56 | 5.80 | C_16_H_12_O_5_ | 284.0685 | 285.0762 | 0.5 | [M-1,3A] 119.0487[C_8_H_7_O]^+^ | +H | Glycitein | GQ | flavonoids |
| 57 | 5.97 | C_27_H_30_O_16_ | 610.1534 | 609.1473 | 1.2 | [M-glc-rha-1.3B] 151.0032[C_7_H_3_O_4_]^-^, [M-glc-rha-OCH_3_] 268.0365[C_15_H_8_O_5_]^-^, [M-glc-rha] 299.0184[C_15_H_7_O_7_]^-^ | -H | Rutin | CH, GC  GQ, MH | flavonoids |
| 58 | 6.10 | C_21_H_20_O_12_ | 464.0955 | 463.0884 | 0.2 | [M-glc] 299.0178 [C_15_H_7_O_7_]^-^, [M-glc-1,3B] 151.0032[C_7_H_3_O_4_]^-^ | -H | Hyperin | CH | flavonoids |
| 58 | 6.11 | C_21_H_20_O_11_ | 448.1006 | 493.1008 | 2.0 | [M-glc] 285.0401 [C_15_H_9_O_6_]^-^, [M-glc-1,3B] 151.0032[C_7_H_3_O_4_]^-^ | +HCOO | Astragalin | GC | flavonoids |
| 60 | 6.20 | C_15_H_10_O_7_ | 302.0427 | 301.0340 | -1.4 | [M-1,3A] 133.0285[C_8_H_5_O_2_]^-^, [M-0,4B] 123.0080[C_6_H_3_O_3_]^-^ | -H | Herbacetin | MH | flavonoids |
| 61 | 6.21 | C_21_H_22_O_9_ | 418.1264 | 417.1187 | -0.4 | [M-glc] 255.0658[C_15_H_11_O_4_]^-^, [M-glc-1,3B] 135.0082[C_7_H_3_O_3_]^-^, [M-0,4A] 323.0755[C_15_H_15_O_8_]^-^ | -H | Liquiritin | GC | flavonoids |
| 62 | 6.23 | C_10_H_10_O_4_ | 194.0597 | 193.0502 | -0.5 | [M-COCH_3_-CH_3_] 135.0082[C_7_H_3_O_3_]^-^, [M-OCOCH_3_] 134.0364[C_8_H_6_O_2_]^-^, [M-COCH_3_] 149.0234[C_8_H_5_O_3_]^-^, [M-CHO-CH_3_] 148.0156[C_8_H_4_O_3_]^-^ | -H | Acetovanillin | CH | esters |
| 63 | 6.39 | C_21_H_20_O_10_ | 432.1057 | 477.1048 | 1.0 | [M-rha-OH] 269.0449[C_15_H_9_O_5_]^-^ | -H, +HCOO | kaeMpferol 7-O-rhaMnoside | HQ | flavonoids |
| 64 | 6.49 | C_7_H_14_O_2_ | 130.0994 | 175.0969 | -0.6 | [M-CH_3_] 116.9278[C_6_H_11_O_2_]^-^ | +HCOO | Methyl hexoate | CH | esters |
| 65 | 6.62 | C_29_H_36_O_15_ | 624.2054 | 623.1977 | -0.4 | [M-C_3_H_6_O_2_] 547.1460[C_26_H_27_O_13_]^-^, [M-C_20_H_30_O_12_] 161.0242 [C_9_H_5_O_3_]^-^, [M-CH_6_O] 589.1557[C_28_H_29_O_14_]^-^, [M-C_9_H_6_O_3_] 461.1660[C_20_H_29_O_12_]^-^ | -H | Forsythiaside | DH | glycosides |
| 66 | 6.66 | C_27_H_32_O_14_ | 580.1792 | 579.1721 | 0.2 | [M-glc-rha] 271.0607[C_15_H_11_O_5_]^-^, [M-glc-rha-1,3B] 151.0031[C_7_H_3_O_4_]^-^ | -H, +HCOO | Naringin | GC | flavonoids |
| 67 | 6.69 | C_27_H_30_O_13_ | 562.1618 | 561.1618 | 0.4 | [M-glc-rib-1,3B] 135.0080 [C_7_H_3_O_3_]^-^ | -H | Glycyroside | GC | flavonoids |
| 68 | 6.80 | C_22_H_22_O_9_ | 430.1264 | 429.1192 | 0.1 | [M-glc] 267.0653[C_16_H_11_O_4_]^-^, [M-glc-OCH_3_] 237.0539[C_15_H_9_O_3_]^-^, [M-glc-CH_3_] 252.0412 [C_15_H_8_O_4_]^-^, [M-glc-1,3B] 135.0078 [C_7_H_3_O_3_]^-^ | -H, HCOO | Ononin | GC | flavonoids |
| 69 | 6.86 | C_16_H_16_O_4_ | 272.1049 | 273.1119 | -0.3 | [M-CH_3_] 258.0887[C_15_H_14_O_4_]^+^, [M-OCH­_2_] 245.1172[C_15_H_17_O_3_]^+^, [M-OCH_3_] 241.0849[C_15_H_13_O_3_]^+^, [M-OCH_2_-CH_3_] 229.0858[C_14_H_13_O_3_]^+^ | +H | Vestitol | GC | others |
| 70 | 7.05 | C_28_H_34_O_15_ | 610.1898 | 609.1827 | 0.2 | [M-glc-rha] 301.0712[C_16_H_13_O_6_]^-^, [M-glc-rha-CH_3_] 286.0476[C15H10O6]^-^, [M-glc-rha-1,3] 151.0031[C_7_H_3_O_4_]^-^, [M-glc-rha-OCH_3_] 271.0605[C_15_H_11_O_5_]^-^, [M-CH_3_-1.3B] 134.0365[C_8_H_6_O_2_]^-^ | -H | Hesperidin | MH | flavonoids |
| 71 | 7.20 | C_15_H_10_O_6_ | 286.0478 | 285.0400 | -0.5 | [M-1,3B] 151.0027[C_7_H_3_O_4_]^-^, [M-0,4B] 107.0130[C_6_H_3_O_2_]^-^ | -H | Kaempferol | CH, GC  MH, RS | flavonoids |
| 72 | 7.23 | C_8_H_14_O | 126.1045 | 125.0966 | -0.6 | [M-2CH_3_] 197.0652[C_6_H_9_O]^-^ | -H | Sulcatone | GZ, MH | ketones |
| 73 | 7.27 | C_10_H_8_O_3_ | 176.0473 | 175.0398 | -0.3 | [M-CH_3_] 160.0158[C_9_H_4_O_3_]^-^ | -H | Ayapanin | CH | coumarins |
| 74 | 7.36 | C_26_H_30_O_13_ | 550.1686 | 549.1615 | 0.1 | [M-glc-api-1,3A] 119.0494[C_8_H_7_O]^-^, [M-api-0,4A] 323.0774[C_15_H_15_O_8_]^-^, [M-glc-api-1,4A] 147.0440[C_9_H_7_O_2_]^-^, [M-glc-0,4B] 227.0940[C_11_H_15_O_5_]^-^ | -H | Liquiritin apioside | GC | flavonoids |
| 75 | 7.38 | C_11_H_10_O_4_ | 206.0579 | 251.0559 | -0.2 | [M-OCH_3_-1,4] 121.0288[C_7_H_5_O_2_]^-^, [M-1,4] 151.0392 [C_8_H_7_O_3_]^-^ | +HCOO | Scoparone | CH | coumarins |
| 76 | 7.42 | C_7_H_6_O_3_ | 138.0317 | 137.0238 | -0.6 | [M-OH-CHO] 93.0339[C_6_H_5_O]^-^, [M-OH] 121.0289[C_7_H_5_O_2_]^--^ | -H | Protocatechualdehyde | GZ | aldehydes |
| 77 | 7.52 | C_15_H_10_O_6_ | 286.0477 | 285.0398 | -0.7 | [M-1,4B] 139.0028[C_6_H_3_O_4_]^-^ | -H | Scutellarein | HQ | flavonoids |
| 78 | 7.58 | C_21_H_22_O_9_ | 418.1264 | 417.1185 | -0.6 | [M-glc-0,4A] 165.0553[C_9_H_9_O_3_]^-^ | -H | Neoliquiritin | GC | flavonoids |
| 79 | 7.59 | C_21_H_22_O_9_ | 418.1264 | 419.1339 | 0.2 | [M-C_9_H_10_O_5_] 221.0808[C_12_H_13_O_4_]^+^, [M-C_6_H_8_O_5_] 259.0965[C_15_H_15_O_4_]^+^, [M-C_11_H_14_O_6_] 177.0546[C_10_H_9_O_3_]^+^, [M-C_6_H_10_O_5_] 257.0808[C_15_H_13_O_4_]^+^ | +H | Isoliquiritin | GC | glycosides |
| 80 | 7.68 | C_17_H_14_O_8_ | 346.0689 | 345.0613 | -0.3 | [M-CH_3_-1,3A] 149.0235[C_8_H_5_O_3_]^-^, [M-1,3A] 163.0393[C_9_H_7_O_3_]^-^ | -H | Viscidulin Ⅲ | HQ | flavonoids |
| 81 | 7.70 | C_10_H_8_O_4_ | 192.0423 | 191.0339 | -1.1 | [M-OCH_3_] 161.0235[C_9_H_5_O_3_]^-^ | -H | Scopoletol | CH, BZ  GC, GQ | coumarins |
| 82 | 7.76 | C_21_H_18_O_11_ | 446.0849 | 445.0776 | -0.1 | [M-glcA] 267.0297[C_15_H_7_O_5_]^-^, [M-glcA-0,4A] 145.0287[C_9_H_5_O_2_]^-^, [M-glcA-1,3A] 101.0397[C_8_H_5_]^-^ | -H | Baicalin | CH, HQ | flavonoids |
| 83 | 7.76 | C_9_H_8_O_2_ | 148.0524 | 193.0494 | -1.2 | [M-COOH] 101.0397[C_8_H_5_]^-^, [M-OH] 131.0498 [C_9_H_7_O]^-^ | +HCOO | Cinnamic acid | GZ | organic acids |
| 84 | 7.76 | C_6_H_8_O_6_ | 176.0321 | 175.0241 | -0.7 | [M-2OH-CH_2_OH] 110.00-2[C_5_H_2_O_3_]^-^, [M-OH] 157.0134[C_6_H_5_O_5_]^-^, [M-OH-2CH_2_OH] 99.0080[C_4_H_3_O_3_]^+^, [M-OH-CH_2_OH] 129.0185[C_5_H_5_O_4_]^-^ | -H | vitamin C | GQ | others |
| 85 | 7.84 | C_15_H_12_O_6_ | 288.0634 | 287.0554 | -0.7 | [M-1,3B] 151.0026[C_7_H_3_O_4_]^-^ | -H | Eriodictyol | MH | flavonoids |
| 86 | 7.87 | C_18_H_16_O_7_ | 344.0896 | 343.0821 | -0.2 | [M-CH_3_-1,3A] 132.0212[C_8_H_4_O_2_]^-^, [M-1,3A] 147.0442[C_9_H_7_O_2_]^-^ | -H | Rivularin | HQ | flavonoids |
| 87 | 7.88 | C_9_H_8_O_3_ | 164.0473 | 209.0457 | 0.1 | [M-OH] 147.0442[C_9_H_7_O_2_]^-^, [M-CH-COOH] 107.0495[C_7_H_7_O]^-^, [M-COOH] 119.0493[C_8_H_7_O]^-^, [M-C_2_H_2_COOH] 93.0339[C_6_H_5_O]^-^ | -H, +HCOO | 2-Coumarate | GZ | organic acids |
| 88 | 7.88 | C_9_H_10_O_3_ | 166.0630 | 165.0556 | -0.2 | [M-2CH_3_] 137.0238[C_7_H_5_O_3_]^-^, [M-OH] 147.0443[C_9_H_7_O_2_]^-^, [M-OCH_3_] 134.0366[C_8_H_6_O_2_]^-^, [M-OH-CH_3_] 132.0212[C_8_H_4_O_2_]^-^ | -H | Paeonol | CH | others |
| 89 | 7.90 | C_31_H_40_O_15_ | 652.2367 | 675.2273 | 1.4 | [M-C_16_H_23_O_9_] 293.1010[C_15_H_17_O_6_]^+^, [M-X_17_H_25_O_9_] 279.0863[C_14_H_15_O_6_]^+^, [M-C_16_H_25_O_10_] 275.0914[C_15_H_15_O_5_]^+^, [M-C_21_H_31_O_12_] 177.0547[C_10_H_9_O_3_]^+^ | +Na | Isomartynoside | DH | glycosides |
| 90 | 8.02 | C_10_H_12_O | 148.0888 | 193.0865 | -0.5 | [M-CHCH_3_] 119.0495[C_8_H_7_O]^-^, [M-CH_3_-C_2_H_2_CH_3_] 91.0184[C_6_H_3_O]^-^, [M-2CH_3_] 117.0338[C_8_H_5_O]^-^ | +HCOO | Anethole | GC, GZ | others |
| 91 | 8.12 | C_8_H_8_O_3_ | 152.0473 | 197.0454 | -0.2 | [M-CHO] 121.0289[C_7_H_5_O_2_]^-^, [M-CH_3_] 138.0311[C_7_H_6_O_3_]^-^,  [M-CHO] 122.0361[C_7_H_6_O_2_]^-^ | +HCOO | 4-Methoxysalicyaldehyde | CH | aldehydes |
| 92 | 8.14 | C_21_H_20_O_11_ | 448.1006 | 447.0936 | 0.3 | [M-glcA] 271.0609[C_15_H_11_O_5_]^-^ | -H | Dihydrobaicalin | HQ | flavonoids |
| 93 | 8.17 | C_10_H_15_NO | 165.1154 | 164.1076 | -0.5 | [M-OH-CH_3_] 130.0654[C_9_H_8_N]^-^ | -H | Hordenine | MH | others |
| 94 | 8.26 | C_15_H_10_O_7_ | 302.0427 | 301.0352 | -0.2 | [M-1,3B] 151.0031[C_7_H_3_O_4_]^-^, [M-1,4B] 123.0080[C_6_H_3_O_3_]^-^ | -H | Quercetin | CH, GC  GQ, MH | flavonoids |
| 95 | 8.26 | C_15_H_20_O_4_ | 264.1362 | 263.1286 | -0.3 | [M-CH_3_-OH-COO] 191.1433[C_13_H_19_O]^-^, [M-OH-COO] 201.1274[C_14_H_17_O]^-^, [M-COO] 219.1380[C_14_H_19_O_2_]^-^ | -H | Vulgarin | RS | esters |
| 96 | 8.28 | C_16_H_12_O_7_ | 316.0583 | 315.0505 | -0.6 | [M-1,3B] 151.0031[C_7_H_3_O_4_]^-^, [M-CH_3_] 300.0265[C_15_H_8_O_7_]^-^, [M-CH_3_-1,3A] 150.0311[C_8_H_6_O_3_]^-^ | -H, +HCOO | Isorhamnetin | CH, GC | flavonoids |
| 97 | 8.42 | C_10_H_8_ | 128.0626 | 173.0600 | -0.8 | [M+HCOO] 173.0600[C_11_H_9_O_2_]^-^ | +HCOO | Naphthalene | CH, GZ  MH | others |
| 98 | 8.44 | C_16_H_14_O_5_ | 286.0841 | 285.0764 | -0.4 | [M-0,4B] 137.0234[C_7_H_5_O_3_]^-^ | -H | Dihydrooroxylin | HQ | flavonoids |
| 99 | 8.44 | C_42_H_64_O_16_ | 824.4194 | 823.4119 | -0.3 | [M-C_30_H_48_O_4_] 351.0569[C_12_H_15_O_12_]^-^, [M-OH] 807.4172[C_42_H_63_O_15_]^-^, [M-O-OH] 793.4380[C_42_H_65_O_14_]^-^, [M-CH_3_-glcA] 633.3647[C_35_H_53_O_10_]^-^ | -H | Licorice-saponin J2 | GC | saponins |
| 100 | 8.72 | C_47_H_76_O_17_ | 944.5345 | 989.5324 | -0.3 | [M-CH_3_-OH] 911.4646[C_46_H_71_O_18_]^-^, [M-rha-CH_3_] 779.4234[C_41_H_63_O_14_]^-^, [M-CH_3_] 927.4959[C_47_H_75_O_18_]^-^, [M-C_17_H_23_O_2_] 653.3390[C_30_H_53_O_15_]^-^ | +HCOO | Saikosaponin K | CH | saponins |
| 101 | 8.85 | C_22_H_20_O_11_ | 460.1006 | 459.0928 | -0.5 | [M-glcA] 283.0605[C_16_H_11_O_5_]^-^, [M-glcA-CH_3_] 268.0371[C_15_H_8_O_5_]^-^, [M-OCH_3_-glcA] 253.0498[C_15_H_9_O_4_]^-^,[M-glcA-OCH_3_-0,4A] 145.0286[C_9_H_5_O_2_]^-^, [M-glcA-0,4B] 137.0233[C_7_H_5_O_3_]^-^ | -H | Wogonoside | CH | flavonoids |
| 102 | 9.04 | C_22_H_22_O_10_ | 446.1213 | 449.1190 | -0.9 | [M-C_7_H_11_O_5_] 270.0515[C_15_H_10_O_5_]^-^, [M-C_13_H_12_O_7_] 165.0548[C_9_H_9_O_3_]^-^, [M-C_7_H_10_O_6_] 255.0648[C_15_H_11_O_4_]^-^, [M-C_4_H_8_O_4_] 325.0711[C_18_H_13_O_6_]^-^ | -H | Trifolirhizin | RS | glycosides |
| 103 | 9.14 | C_15_H_12_O_5_ | 272.0685 | 271.0604 | -0.8 | [M-1,3B] 151.0028[C_7_H_3_O_4_]^-^, [M-0,4B] 107.0131[C_6_H_3_O_2_]^-^ | -H | Naringenin | GC, MH | flavonoids |
| 104 | 9.36 | C_12_H_14_O_4_ | 222.0892 | 221.0809 | -1.1 | [M-C_2_H_5_] 193.0494[C_10_H_9_O_4_]^-^, [M-C_6_H_4_COOH] 99.0444[C_5_H_7_O_2_]^-^,[M-OH-C_3_H_7_] 161.0229[C_9_H_5_O_3_]^-^, [M-COOH-C_2_H_5_] 146.0360[C_9_H_6_O_2_]^-^ | -H | Monobutyl phthalate | MH | esters |
| 105 | 9.38 | C_15_H_14_O_2_ | 226.0994 | 227.1060 | -0.7 | [M-C_6_H_5_CH_2_] 137.0597[C_8_H_9_O_2_]^+^, [M-OC_2_H_4_C_6_H_5_] 107.0491[C_7_H_7_O]^+^, [M-C_2_H_4_C_6_H_5_] 122.0362[C_7_H_6_O_2_]^+^ | +H | Phenethyl benzoate | GZ | esters |
| 106 | 9.39 | C_16_H_12_O_6_ | 300.0634 | 299.0555 | -0.6 | [M-OCH_3_] 269.0446[C_15_H_9_O_5_]^-^, [M-CH_3_] 284.0319[C_15_H_8_O_6_]^-^, [M-1,3B] 151.0025[C_7_H_3_O_4_]^-^, [M-CH_3_-1,3A] 132.0205[C_8_H_4_O_2_]^-^ | -H, +HCOO | Diosmetin | GC | flavonoids |
| 107 | 9.44 | C_10_H_12_O | 148.0888 | 193.0859 | -1.1 | [M-CH_3_] 131.0500[C_9_H_7_O]^-^ | +HCOO | m-Ethylacetophenone | MH | ketones |
| 108 | 9.58 | C_42_H_72_O_14_ | 800.4922 | 845.4902 | -0.2 | [M-glc] 637.4316[C_36_H_61_O_9_]^-^, [M-glc-glc] 475.3787[C_30_H_51_O_4_]^-^, [M-glc-glc-R] 391.2838[C_24_H_39_O_4_]^-^, [M-CH_3_] 784.4615[C_41_H_68_O_14_]^-^ | -H, +HCOO | Ginsenoside rf | RS | saponins |
| 109 | 9.68 | C_33_H_45_NO_10_ | 615.3044 | 616.3115 | -0.1 | [M-C_2_H_4_O_2_] 556.2905[C_31_H_42_NO_8_]^+^, [M-C_3_H_8_O_3_] 524.2643[C_30_H_38_NO_7_]^+^, [M-CH_4_O] 584.2854[C_32_H_42_NO_9_]^+^, [M-C_7_H_6_O] 496.2667[C_26_H_40_O_9_]^+^ | +H | Hypaconitine | GQ | alkaloids |
| 110 | 9.85 | C_42_H_68_O_13_ | 770.4816 | 815.4808 | 0.9 | [M-ara] 637.4310 [C_36_H_61_O_9_]^-^, [M-glc-ara] 475.3786[C_30_H_51_O_4_]^-^, [M-OH] 761.4482[C_42_H_65_O_12_]^-^, [M-2CH_2_OH-rha-glc] 409.3112[C_28_H_41_O_2_]^-^ | -H, +HCOO | Saikosaponin b1 | CH | saponins |
| 111 | 9.85 | C_41_H_70_O_13_ | 770.9909 | 815.4808 | 0.9 | [M-OH-CH_3_-C_5_H_9_-glc-xyl] 369.2435[C_24_H_33_O_3_]^-^, [M-xyl] 619.3852[C_35_H_55_O_9_]^-^, [M-2OH-C_4_H_7_] 679.4063[C_37_H_59_O_11_]^-^, [M-glc-xyl] 475.3793[C_30_H_51_O_4_]^-^ | -H, +HCOO | notoginsenoside R2 | RS | saponins |
| 112 | 9.94 | C_15_H_10_O_5_ | 270.0528 | 269.0451 | -0.5 | [M-0,4A] 145.0286[C_9_H_5_O_2_]^-^, [M-1,3A] 101.0393[C_8_H_5_]^-^, [M-1,4A] 129.0345[C_9_H_5_O]^-^ | -H | Baicalein | HQ | flavonoids |
| 113 | 10.07 | C_17_H_14_O_6_ | 314.0790 | 359.0770 | -0.3 | [M-1,3B] 167.0342[C_8_H_7_O_4_]^-^, [M-0,4B] 121.0286 [C_7_H_5_O_2_]^-^ | +HCOO | Jaranol | GC | flavonoids |
| 114 | 10.13 | C_48_H_76_O_19_ | 956.4981 | 955.4914 | 0.6 | [M-glc] 793.4377[C_42_H_65_O_14_]^-^, [M-COOglc] 749.4462[C_41_H_65_O_12_]^-^, [M-glcA-2glc] 453.3360[C_30_H_45_O_3_]^-^ | -H | Ginsenoside Ro | RS | saponins |
| 115 | 10.22 | C_11_H_20_O_2_ | 184.1463 | 229.1438 | -0.8 | [M-COCH­_2_] 139.1122[C_9_H_15_O]^-^, [M-COC_2_H_4_] 127.1122[C_8_H_15_O]^-^, [M-O]167.1434[C_11_H_19_O]^-^, [M-COC_2_H­_4_-C_2_H_5_] 99.0809[C_6_H_11_O]^-^ | +HCOO | Undecan-4-olide | CH | aldehydes |
| 116 | 10.29 | C_42_H_62_O_17_ | 838.3987 | 837.3919 | 0.4 | [M-2glcA] 485.3262[C_30_H_45_O_5_]^-^ | -H | Licorice-saponin G2 | GC | saponins |
| 117 | 10.30 | C_28_H_46_O | 398.3549 | 421.3453 | 1.2 | [M-C_9_H_11_] 187.1481[C_14_H_19_]^+^, [M-C_12_H_25_O] 213.1638[C_16_H_21_]^+^, [M-C_7_H_19_O] 279.2107[C_21_H_27_]^+^, [M-C_9_H_23_O] 251.1794[C_19_H_23_]^+^ | +Na | Ostreasterol | GQ | others |
| 118 | 10.33 | C_48_H_80_O_17_ | 928.5396 | 927.5380 | 0.2 | [M-rha] 781.4734[C_42_H_69_O_13_]^-^, [M-glc] 765.4788[C_42_H_69_O_12_]^-^, [M-rha-glc] 619.4204[C_36_H_59_O_8_]^-^, [M-2glc-rha] 457.3671[C_30_H_49_O_3_]^-^ | -H | Saikosaponin f | CH | saponins |
| 119 | 10.36 | C_13_H_26_O_2_ | 214.1933 | 259.1908 | -0.7 | [M-OCH_3_-C_3_H_7_] 139.1122[C_9_H_15_O]^-^, [M-OCH_3_-C_4_H_9_] 127.1121[C­_8_H_15_O]^-^, [M-OCH_3_-C_6_H_13_]^-^, [M-C_4_H_9_] 157.1224[C_9_H_17_O_2_]^-^ | +HCOO | Methyl laurate | CH, HQ  MH | esters |
| 120 | 10.39 | C_16_H_12_O_4_ | 268.0736 | 267.0658 | -0.5 | [M-CH_3_] 252.0421[C_15_H_8_O_4_]^-^, [M-1,3B] 135.0079[C_7_H_3_O_3_]^-^ | -H | Formononetin | GC | flavonoids |
| 121 | 10.49 | C_11_H_14_O | 162.1045 | 161.0911 | -0.6 | [M-C_3_H_7_] 119.0494[C_8_H_7_O]^-^ | -H | Valerophenone | CH | ketones |
| 122 | 10.52 | C_12_H_12_O_2_ | 188.0837 | 189.0906 | -0.4 | [M-CH_3_-COO] 128.0620[C_10_H_8_]^+^, [M-C_2_H_5_-COO] 115.0542[C_9_H_7_]^+^, [M-COO] 143.0855[C_11_H_11_]^+^, [M-CH_3_] 173.0597[C_11_H_9_O_2_]^+^ | +H | 3-Butylidenephthalide | CH | esters |
| 123 | 10.53 | C_9_H_18_O_2_ | 158.1307 | 181.1217 | 1.8 | [M-CH_3_] 128.0614[C_8_H_15_O]^+^ | +Na | Methyl octylate | CH, HQ  MH | esters |
| 124 | 10.61 | C_11_H_16_O | 164.1201 | 163.1123 | -0.5 | [M-H] 163.1123[C_11_H_15_O]^-^ | -H, +HCOO | O-Methylthymol | CH | others |
| 125 | 10.61 | C_12_H_16_O_3_ | 208.1100 | 207.1021 | -0.6 | [M-CH_3_] 191.0721[C_11_H_11_O_3_]^-^, [M-CH_3_-C_2_H_2_CH_3_] 149.0236[C_8_H_5_O_3_]^-^, [M-2CH_3_-OCH_3_] 149.0605[C_9_H_9_O_2_]^-^, [M-OCH_3_-C_2_H_2_CH_3_] 137.0599[C_8_H_9_O_2_]^-^ | -H | beta-asarone | GZ | others |
| 126 | 10.81 | C_42_H_62_O_16_ | 822.4038 | 821.3969 | 0.4 | [M-glcA] 645.3646[C_36_H_53_O_10_]^-^, [M-2glcA] 469.3317[C_30_H_45_O_4_]^-^, [M-COOH-glcA] 601.3743[C_35_H_53_O_8_]^-^ | -H | Licorice-saponin K2 | GC | saponins |
| 127 | 10.83 | C_42_H_62_O_16_ | 822.4038 | 821.3969 | 0.4 | [M-C_30_H_46_O_4_] 351.0566[C_12_H_15_O_12_]^-^, [M-C_7_H_6_O_6_] 635.3812[C_35_H_55_O_10_]^-^, [M-C_6_H_6_O_7_] 631.3856[C_36_H_55_O_9_]^-^, [M-CH_2_O_3_] 759.3966[C_41_H_59_O_13_]^-^ | -H | Glycyrrhizic acid | GC | saponins |
| 128 | 10.86 | C_12_H_14_O_3_ | 206.0943 | 205.0865 | -0.8 | [M-C_2_H_2_COOC_2_H­_5_] 106.0416[C_7_H_6_O]^-^, [M-COOC_2_H_5_] 131.0500[C_9_H_7_O]^--^ | -H | Ethyl methoxycinnamate | GZ | esters |
| 129 | 10.91 | C_15_H_10_O_5_ | 270.0528 | 269.0453 | -0.3 | [M-1,3A] 117.0335[C_8_H_5_O]^-^ | -H, +HCOO | Apigenin | HQ, MH | flavonoids |
| 130 | 11.41 | C_16_H_12_O_5_ | 284.0685 | 283.0608 | -0.4 | [M-CH_3_] 268.0372[C_15_H_8_O]^-^ | -H | Genkwanin | MH | flavonoids |
| 131 | 11.48 | C_11_H_10_O | 158.0732 | 203.0708 | -0.6 | [M-CH_3_] 143.0504[C_10_H_7_O]^-^ | +HCOO | 2-Methyl-1-naphthol | MH | others |
| 132 | 11.69 | C_42_H_68_O_13_ | 780.4660 | 825.4639 | -0.3 | [M-rha] 633.4007[C_36_H_57_O_9_]^-^ | -H, +HCOO | Saikosaponin a | CH | saponins |
| 133 | 11.73 | C_19_H_18_O_8_ | 374.1002 | 373.0925 | -0.4 | [M-1,3A] 147.0441[C_9_H_7_O_2_]^-^, [M-CH_3_-1,3A] 132.0208[C_8_H_4_O_2_]^-^ | -H | Skullcapflavone II | HQ | flavonoids |
| 134 | 11.79 | C_10_H_10_O_2_ | 162.0681 | 207.0653 | -1.0 | [M-C_2_H_2_] 135.0440[C_8_H_7_O_2_]^-^, [M-CH_2_-C_2_H_2_] 121.0284[C_7_H_5_O_2_]^-^, [M-CH] 149.06015[C_9_H_9_O_2_]^-^, [M-O] 143.0497[C_10_H_7_O]^-^ | +HCOO | Safrol | GZ, GQ | others |
| 135 | 11.83 | C_16_H_12_O_5_ | 284.0685 | 283.0605 | -0.7 | [M-CH_3_] 268.0370[C_15_H_8_O_5_]^-^, [M-OCH_3_] 253.0491[C_15_H_9_O_4_]^-^ | -H | Wogonin | HQ | flavonoids |
| 136 | 11.83 | C_16_H_12_O_5_ | 284.0685 | 283.0605 | -0.7 | [M-CH_3_] 268.0371[C_15_H_8_O_5_]^-^, [M-OCH_3_] 253.0498[C_15_H_9_O_4_]^-^, [M-CH_3_-C_7_H_6_O] 163.0029[C_8_H_3_O_4_]^-^, [M-CH_3_-OCH_3_] 239.0342[C_14_H_7_O_4_]^-^ | -H | Physcion | MH | others |
| 137 | 11.99 | C_16_H_12_O_5_ | 284.0685 | 283.0603 | -0.9 | [M-OCH_3_] 253.0497[C_15_H_9_O_4_]^-^, [M-CH_3_] 268.0367[C_15_H_8_O_5_]^-^, [M-1,3A] 131.0493[C_9_H_7_O]^-^ | -H | Acacetin | HQ | flavonoids |
| 138 | 12.17 | C_41_H_68_O_13_ | 782.4816 | 805.4724 | 1.6 | [M-C_11_H_23_O_11_] 437.3414[C_30­_H_45_O_2_]^+^, [M-C_6_H_15_O_6_] 585.3786[C_35_H_53_O_7_]^+^, [M-glc-C_5_H_9_O_4_] 455.3510[C_30_H_47_O_3_]^+^, [M-C_27_H_49_O_6_] 187.1481[C_14_H_19_]^+^ | +Na | Ginsenoside La | RS | saponins |
| 139 | 12.20 | C_16_H_8_O_4_ | 264.0423 | 309.0399 | -0.6 | [M-C_6_H_4_O] 173.0233[C_10_H_5_­O_3_]^-^, [M-C_7_H_4_O_2_] 145.0286[C_9_H_5_O_2_]^-^, [M-O] 248.0465[C_16_H_8_O_3_]^-^, [M-C_3_O_2_] 195.0437[C_13_H_7_O_2_]^-^ | +HCOO | Frutinone A | RS | ketones |
| 140 | 12.21 | C_24_H_32_O_7_ | 432.2148 | 455.2038 | -0.2 | [M-2OH-CH_3_] 384.1931[C_23_H_28_O_5_]^+^, [M-OH] 415.2115[C_24_H_31_O_6_]^+^, [M-2OH-2CH_3_] 369.1696[C_22_H_25_O_5_]^+^, [M-COCH_3_-2OH-CH_3_] 338.1513[C_21_H_22_O_4_]^+^ | +H, +Na | malkangunin | RS | esters |
| 141 | 12.21 | C_20_H_41_Cl_3_Si | 414.2043 | 415.2112 | -0.3 | [M-C_6_H_13_] 331.1177[C_14_H_30_Cl_3_Si]^+^, [M-CH_3_] 400.1881[C_19_H_39_Cl_3_Si]^+^, [M-C_4_H_9_] 359.1490[C_16_H_34_Cl_3_Si]^+^, [M-C_7_H_15_] 314.0786[C_13_H_25_Cl_3_Si]^+^ | +H | Trichloroicosylsilane | MH | others |
| 142 | 12.28 | C_42_H_64_O_15_ | 808.4245 | 807.4183 | 1.0 | [M-2glcA] 455.3544[C_30_H_47_O_3_]^-^ | -H | Licorice-saponin B2 | GC | saponins |
| 143 | 12.34 | C_17_H_14_O_6_ | 314.0790 | 313.0714 | -0.4 | [M-CH_3_] 298.0470[C_16_H_10_O_6_]^-^, [M-CH_3_-OCH_3_] 268.0361[C_15_H_8_O_5_]^-^, [M-OCH_3_] 283.0626[C_16_H_11_O_5_]^-^, [M-CH_3_-1,3A] 117.0335[C_8_H_5_O]^-^ | -H | Pectolinarigenin | MH | flavonoids |
| 144 | 12.34 | C_11_H_12_O_2_ | 176.0837 | 177.0902 | -0.8 | [M-CHCH_2_OCOCH_3_] 91.0542[C_7_H_7_]^+^, [M-OCOCH_3_] 117.0699[C_9_H_9_]^+^, [M-CH_2_OCOCH_3_] 103.0542[C_8_H_7_]^+^, [M-COCH_3_] 135.0804[C_9_H_11_O]^+^ | +H | Cinnamyl acetate | GZ | esters |
| 145 | 12.34 | C_10_H_12_O_3_ | 180.0786 | 181.0852 | -0.7 | [M-OH-CH_3_] 151.0754[C_9_H_11_O_2_]^+^, [M-COOH-CH_3_] 121.0648[C_8_H_9_O]^+^, [M-COOH-OCH_3_] 103.0542[C_8_H_7_]^+^, [M-COOH] 135.0804[C_9_H_11_O]^+^ | +H | 3,5-Dimethyl-p-anisic acid | RS | organic acids |
| 146 | 12.34 | C_12_H_16_O_3_ | 108.1100 | 209.1168 | -0.4 | [M-2CH_3_] 179.0702[C_10_H_11_O_3_]^+^, [M-OCH_3_-C_2_H_2_] 151.0754[C_9_H_11_O_2_]^+^, [M-CH_3_-OCH_3_-CH_2_C_2_H_2_] 123.0441[C_7_H_7_O_2_]^+^, [M-2CH_3_-OCH_3_] 147.0441[C_9_H_7_O_2_]^+^ | +H | Elemicin | RS | others |
| 147 | 12.36 | C_10_H_18_O | 154.1358 | 177.1264 | 1.4 | [M-CH_3_] 105.0694[C_8_H_9_]^+^, [M-CH_3_-C_2_H_2_] 91.0542[C_7_H_7_]^+^, [M-C_2_H_2_] 107.0855[C_8_H_11_]^+^, [M-CH_2_CH_3_CH_3_] 93.0699[C_7_H_9_]^+^ | +Na | cis-Piperitol | MH | others |
| 148 | 12.39 | C_10_H_14_ | 134.1096 | 157.1005 | 1.7 | [M-C_2_H_2_] 105.0699[C_8_H_9_]^+^, [M-CH_3_] 119.0849[C_9_H_11_]^+^, [M-C_3_H_5_] 91.0542[C_7_H_7_]^+^, [M-2CH_3_] 103.0542[C_8_H_7_]^+^ | +H, +Na | p-Mentha-1,3,8-triene | MH | others |
| 149 | 12.45 | C_8_H_8_ | 104.0626 | 105.0694 | -0.5 | [M-CH_2_] 91.0538[C_7_H_7_]^+^, [M-CHCH_2_] 77.0386[C_6_H_5_]^+^ | +H | Styrene | GZ, MH | others |
| 150 | 12.46 | C_12_H_16_O_2_ | 192.1150 | 193.1220 | -0.3 | [M-C_4_H_9_] 137.0597[C_8_H_9­_O_2_]^+^, [M-COOC_5_H_11_] 77.0386[C_6_H_5_]^+^, [M-C_3_H_7_] 149.0597[C_9_H_9_O_2_]^+^, [M-COC_5_H_11_] 105.0335[C_7_H_5_O]^+^ | +H, +Na | Isoamyl benzoate | GZ | esters |
| 151 | 12.46 | C_8_H_8_O_2_ | 136.0524 | 137.0594 | -0.3 | [M-COOH] 91.0538[C_7_H_7_]^+^, [M-CH_2_COOH] 77.0386[C_6_H_5_]^+^, [M-OH] 119.0491[C_8_H_7_O]^+^ | +H | Phenylacetic acid | GZ | organic acids |
| 152 | 12.46 | C_26_H_32_O_5_ | 424.2250 | 425.2339 | 1.6 | [M-C_9_H_20_] 297.0758[C_17_H_13_O_5_]^+^, [M-C_11_H_22_] 271.0601[C_15_H_11_O_5_]^+^, [M-C_18_H_24_O_3_] 137.0597[C_8_H_9_O_2_]^+^, [M-C_10_H_22_] 283.0601[C_16_H_11_O_5_]^+^ | +H | Licoricidin | GC | others |
| 153 | 12.48 | C_47_H_74_O_18_ | 926.4875 | 925.4812 | 1.0 | [M-Oglc] 745.4156[C_41_H_61_O_12_]^-^, [M-rib-glc] 629.3696[C_36_H_53_O_9_]^-^, [M-glcA-rib-glc] 455.3544[C_30_H_47_O_3_]^-^ | -H | Araloside A | RS | saponins |
| 154 | 12.48 | C_19_H_14_NO_4_ | 320.0923 | 343.0816 | 0.1 | [M-C_11_H_5_NO_2_] 137.0597[C_8_H_9_O_2_]^+^, [M-C_10_H_5_NO_2_] 149.0593[C_9_H_9_O_2_]^+^, [M-C_11_H_7_NO_2_] 135.0441[C_8_H_7_O_2_]^+^ | +Na | Coptisine | HQ | alkaloids |
| 155 | 12.57 | C_15_H_18_ | 198.1409 | 199.1479 | -0.2 | [M-CH_3_-C_3_H_7_] 141.0699[C_11_H_9_]^+^, [M-2CH_3_-C_3_H_7_] 128.0620[C_10_H_8_]^+^, [M-[M-C_3_H_7_] 157.1012[C_12_H_13_]^+^, [M-CH_3_] 169.1012[C_13_H_13_]^+^ | +H | Azulol | HQ, MH | others |
| 156 | 12.57 | C_9_H_12_O | 135.0888 | 137.0956 | -0.5 | [M-CH_2_OH] 105.0696[C_8_H_9_]^+^, [M-OH] 119.0855[C_9_H_11_]^+^, [M-C_2_H_4_OH] 91.0542[C_7_H_7_]^+^, [M-C_3_H_6­_OH] 77.0386[C_6_H_5_]^+^ | +H | Benzenepropanol | GZ | others |
| 157 | 12.57 | C_10_H_12_ | 132.0939 | 133.1009 | -0.3 | [M-C_2_H_2_] 105.0696[C_8_H_9_]^+^, [M-CH_3_]119.0855[C_9_H_11_]^+^, [M-C_2_H_2_-CH_3_] 91.0542[C_7_H_7_]^+^, [M-CH_3_] 103.0542[C_8_H_7_]^+^ | +H | 2,3-Dimethylstyrene | CH | others |
| 158 | 12.57 | C_9_H_12_ | 120.0939 | 121.1006 | -0.5 | [M-CH_3_] 105.0696[C_8_H_9_]^+^, [M-2CH_3_] 91.0542[C_7_H_7_]^+^ | +H, +Na | Mesitylene | MH | others |
| 159 | 12.67 | C_42_H_68_O_12_ | 764.4711 | 809.4694 | 0.1 | [M-glc] 601.4106[C_36_H_57_O_7_]^-^ | -H, +HCOO | Saikosaponin e | CH | saponins |
| 160 | 12.82 | C_20_H_20_O_5_ | 340.1311 | 339.1234 | -0.4 | [M-R] 269.0446[C_15_H_9_O_5_]^-^, [M-R-1,3B] 151.0027[C_7_H_3_O_4_]^-^, [M-R-1,3A] 163.0386[C_9_H_7_O_3_]^-^, | -H | Glepidotin B | GC | flavonoids |
| 161 | 12.82 | C_7_H_6_O_3_ | 138.0312 | 139.0387 | -0.3 | [M-OH] 121.0279[C_7_H_5_O_2_]^+^, [M-COOH] 93.0335[C_6_H_5_O]^+^, [M-OH-COOH] 77.0386[C_6_H_5_]^+^ | +H | 4-Oxoniobenzoate | GZ, MH | organic acids |
| 162 | 12.82 | C_7_H_6_O_2_ | 122.0368 | 123.0437 | -0.4 | [M-CHO] 93.0335[C_6_H_5_O]^+^, [M-CHO-OH] 77.0386[C_6_H_5_]^+^, | +H | Salicylaldehyde | GZ, MH | aldehydes |
| 163 | 13.13 | C_15_H_18_O_3_ | 246.1256 | 245.1177 | -0.6 | [M-C_9_H_10_O] 111.0441 [C_6_H_7_O_2_]^-^ | -H | Zederone | GQ | ketones |
| 164 | 13.29 | C_16_H_14_O_4_ | 270.0892 | 271.0966 | 0.1 | 147.0439[C_9_H_7_O_2_]^+^ | +H,+Na | Echinatin | GC | flavonoids |
| 165 | 13.59 | C_28_H_34_O_9_ | 514.2203 | 515.2271 | -0.5 | [M-C_16_H_20_O_5_] 221.0808[C_12_H_13_O_4_]^+^, [M-C_13_H_18_O_5_] 259.0965[C_15_H_15_O_4_]^+^, [M-C_18_H_24_O_6_] 177.0546[C_10_H_9_O_3_]^+^, [M-C_18_H_24_O_7_] 161.0597[C_10_H_9_O_2_]^+^ | +H | Gomisin B | RS | esters |
| 166 | 13.68 | C_12_H_18_O_2_ | 194.1307 | 195.1377 | -0.3 | [M-CHO-OH] 149.1325[C_11_H_17_]^+^, [M-O] 177.1274[C_12_H_17_O]^+^, [M-O-C_3_H_7_] 137.0961[C_9_H_13_O]^+^, [M-C_7_H_12­_] 97.0284[C_5_H_5_O_2_]^+^ | +H, +Na | Neocnidilide | RS | esters |
| 167 | 13.68 | C_11_H_16_ | 148.1252 | 149.1321 | -0.4 | [M-C_3_H_7_] 105.0699[C_8_H_9_]^+^, [M-CH_3_] 135.1168[C_10_H_15_]^+^, [M-C_4_H_9_] 93.0699[C_7_H_9_]^+^, [M-C_5_H_11_] 79.0542[C_6_H_7_]^+^ | +H | Amylbenzene | CH | others |
| 168 | 13.72 | C_17_H_32_O_2_ | 268.2402 | 313.2380 | -0.4 | [M-OH-C_9_H_16_] 127.1121[C_8_H_15_O]^-^, [M-C_8_H_16_] 155.1069[C_9_H_15_O_2_]^-^, [M-C_7_H_18_] 165.0909[C_10_H_13_O_2_]^-^, [M-C_8_H_16_O] 139.1119[C_9­_H_15_O]^-^ | +HCOO | Methyl palmitoleate | DH | esters |
| 169 | 13.75 | C_12_H_14_O_2_ | 190.0994 | 191.1066 | -0.1 | [M-COO-C_2_H_5_] 117.0699[C_9_H_9_]^+^, [M-C_5_H_6_O_2_] 91.0542[C_7_H_7_]^+^, [M-COO-C_3_H_7_] 105.0699[C_8_H_9_]^+^, [M-COO] 145.1012[C_11_H_13_]^+^ | +H | cis-ligustilide | CH | esters |
| 170 | 13.89 | C_20_H_20_O_4_ | 324.1362 | 369.1342 | -0.2 | [M-R-0,4A] 147.0441[C_9_H_7_O_2_]^-^, [M-0,4B] 177.0911[C_11_H_13_O_2_]^-^, [M-R-1,3B] 151.0026 [C_7_H_3_O_4_]^-^ | +HCOO | Glabranin | GC | flavonoids |
| 171 | 14.41 | C_13_H_18_O | 190.1358 | 213.1268 | 1.9 | [M-CH_3_-C_4_H_7_O] 105.0692[C_8_H_9_]^+^, [M-2CH_3_] 159.0804[C_11_H_11_O]^+^, [M-CH_3_-C_2_H_5_] 145.0648[C_10_H_9_O]^+^, [M-C_2_H_5_] 161.0961[C_11_H_13_O]^+^ | +H | Damascenone | MH | ketones |
| 172 | 14.80 | C_15_H_26_O | 222.1984 | 267.1960 | -0.6 | [M-C_6_H_13_] 137.0963[C_9_H_13_O]^-^, [M-C_5_H_9_] 151.1117[C_10_H_15_O]^-^ | +HCOO | Juniper camphor | BZ | others |
| 173 | 15.44 | C_30_H_44_O_5_ | 484.3189 | 483.3118 | 0.2 | [M-C_8_H_20_] 367.1542[C_22_H_23_O_5_]^-^, [M-OH] 465.2999[C_30_H_41_O_4_]^-^, [M-COOH-OH] 421.3098[C_29_H_41_O_2_]^-^, [M-C_9_H_20_] 355.1539[C_21_H_23_O_5_]^-^ | -H, +HCOO | Poricoic acid B | FL | organic acids |
| 174 | 15.80 | C_30_H_48_O_4_ | 472.3553 | 471.3479 | -0.1 | [M-C_12_H_16_O_2_] 279.2326[C_18_H_31_O_2_]^-^, [M-C_11_H_14_O_2_] 293.2480[C_19_H_33_O_2_]^-^, [M-COOH] 423.3264[C_29_H_43_O_2_]^-^, [M-C_12_H_14_O_2_] 281.2475[C_18_H_33_O_2_]^-^ | -H, +HCOO | Maslinic acid | MH | organic acids |
| 175 | 15.86 | C_20_H_30_O_4_ | 334.2144 | 333.2066 | -0.5 | [M-C_3_H_7_-OC_4_H_9_] 216.0781[C_13_H_12_O_3_]^-^, [M-C_7_H_15_-OC_4_H_9_] 161.0229[C_9_H_5_O_3_]^-^, [M-C_6_H_13_-C_4_H_9_] 193.0497[C_10_H_9_O_4_]^-^, [M-COOC_8_H_17_] 177.0911[C_11_H_13_O_2_]^-^ | -H | Butyl octyl phthalate | MH | esters |
| 176 | 16.01 | C_31_H_46_O_5_ | 498.3345 | 497.3276 | 0.3 | [M-C­_13_H_16_O_2_] 293.2118[C_18_H_29_O_3_]^-^, [M-C_13_H_16_O_3_] 277.21169[C_18_H_29_O_2_]^-^, [M-C_3_H_6_O_2_] 423.2905[C_28_H_39_O_3_]^-^, [M-C_22_H_30_O_2_] 171.1021[C_9_H_15_O_3_]^-^ | -H, +HCOO | Poricoic acid A | FL | organic acids |
| 177 | 16.41 | C_30_H_46_O_4_ | 480.3396 | 469.3326 | 0.2 | [M-COOH] 425.3417[C_29_H_45_O_2_]^-^ | -H, +HCOO | 18β-Glycyrrhetinic Acid | GC | organic acids |
| 178 | 18.08 | C_30_H_48_O_3_ | 456.3604 | 455.3520 | -1.1 | [M-C_12_H_20_O] 275.2003[C_18_H_27_O_2_]^-^, [M-C_22_H_41_O] 134.0364[C_8_H_6_O_2_]^-^, [M-C_11_H_18_O_2_] 273.2205[C_19_H_29_O]^-^, [M-C_15_H_20_O_2_] 223.2055[C_15_H_27_O]^-^ | -H | Mairin | GC | organic acids |
| 179 | 18.09 | C_14_H_28_O | 212.2140 | 257.2112 | -1.0 | [M-H] 277.2112[C_14_H_27_O]^-^ | +HCOO | 2-Tetradecanone | GC | ketones |
| 180 | 18.88 | C_33_H_52_O_5_ | 528.3815 | 527.3746 | 0.4 | [M-OCOC_2_H_5_] 455.3528[C_30_H_47_O_3_]^-^, [M-C_15_H_22_O_3_] 277.2170[C_18_H_29_O_2_]^-^, [M-C_15_H_20_O_3_] 279.2326[C_18_H_31_O_2_]^-^, [M-OCOCH_3_] 465.3370[C_31_H_45_O_3_]^-^ | -H, +HCOO | pachymic acid | FL | organic acids |
| 181 | 18.95 | C_18_H_30_O_2_ | 278.2246 | 277.2172 | -0.1 | [M-OH-CHC_2_H_5_] 221.1904[C_15_H_25_O]^-^, [M-OH] 259.2060[C_18_H_27_O]^-^, [M-C_10_H_16_O] 125.0964[C_8_H_13_O]^-^, [M-OH-C_5_H_9_] 193.1586[C_13_H_21_O]^-^ | -H, +HCOO | linolenic acid | MH | organic acids |
| 182 | 19.05 | C_14_H_26_ | 194.2034 | 239.2010 | -0.6 | [M+HCOO] 239.2010[C_15_H_27_O_2_]^-^ | +HCOO | 7-Tetradecyne | RS | others |
| 183 | 19.31 | C_14_H_28_O_2_ | 228.2089 | 227.2011 | -0.5 | [M-H] 227.2011[C_14_H_27_O_2_]^-^ | -H | Myristic acid | CH, GQ  HQ, MH | organic acids |
| 184 | 19.50 | C_16_H_33_NO | 255.2562 | 256.2638 | 0.3 | [M-C_11_H_23_] 102.0913[C_5_H_12_NO]^+^, [M-C_12_H_25_] 88.0757[C_4_H_10_NO]^+^, [M-C_10_H_21_] 116.1070[C_6_H_14_NO]^+^, [M-C_4_H_9_-C_4_H_8_CONH­_2_] 97.1012[C_7_H_13_]^+^ | +H | Hexadecanamide | CH | others |
| 185 | 19.78 | C_16_H_30_O_2_ | 254.2246 | 253.2171 | -0.2 | [M-OH-C_2_H_5_] 205.1588[C_14_H_21_O]^-^, [M-OH-C_9_H_19_] 253.2171[C_16_H_29_O_2_]^-^ | -H | zoomaric acid | DH | organic acids |
| 186 | 20.13 | C_10_H_18_O_3_ | 186.1256 | 185.1176 | -0.8 | [M-CH_3_] 171.10181[C_9_H_15_O_3_]^-^, [M-CH_2_CHO-OCH_3_] 109.0651[C_7_H_9_O]^-^, [M-CH_2_COOCH_3_] 113.0964[C_7_H_13_O]^-^, [M-OCH_3_] 155.1068[C_9_H_15_O_2_]^-^ | -H | Methyl 9-oxononanoate | HQ | esters |
| 187 | 20.13 | C_18_H_30_O | 262.2297 | 307.2274 | -0.5 | [M-C_4_H_7_] 205.1586[C_14_H_21_O]^-^, [M-2CH­_3_-C_6_H_11_] 149.0962[C_10_H_13_O]^-^, [M-C_11_H_21_] 109.0651[C_7_H_9_O]^-^, [M-CH_3_-C_6_H_11_] 163.1117[C_11_H_15_O]^-^ | -H,  +HCOO | Farnesylacetone | CH, GQ  MH | ketones |
| 188 | 20.13 | C_8_H_14_O | 126.1045 | 171.1020 | -0.7 | [M-CH_3_] 109.0651[C_7_H_9_O]^-^, [M-CH_2_] 113.0964[C_7_H_13_O]^-^, [M-C_2_H_5_] 97.0650[C_6_H_9_O]^-^ | +HCOO | Vinyl amyl ketone | CH, MH | ketones |
| 189 | 20.19 | C_18_H_32_O_2_ | 280.2402 | 279.2327 | -0.3 | [M-OH] 261.2219[C_18_H_29_O]^-^, [M-OH-C_10_H_17_] 125.0963 [C_8_H_13_O]^-^, [M-OH-C_4_H_9­_] 205.1586[C_14_H_21_O]^-^, [M-OH-C_8_H_12_] 149.0962[C_10_H_13_O]^-^ | -H, +HCOO | Linoleic | CH, GQ  RS | organic acids |
| 190 | 20.30 | C_15_H_30_O_2_ | 242.2246 | 241.2165 | -0.8 | [M-C_2_H_5_-CH_3_] 195.1383[C_12_H_19_O_2_]^-^ | -H | Methyl myristate | CH, HQ  RS | esters |
| 191 | 20.79 | C_36_H_62_O_9_ | 638.4394 | 637.4326 | 0.5 | [M-C_17_H_28­_O_7_] 293.2478[C_19_H_33_O_2_]^-^, [M-C_22_H_34_O_7_] 227.2008[C_14_H_27_O_2_]^-^, [M-C_18_H_30_O_3_] 343.2122[C_18_H_31_O_6_]^-^. [M-C_4_H_8­_O_5_] 501.3938[C_32_H_53_O_4_]^-^ | -H | Ginsenoside rh1 | RS | saponins |
| 192 | 20.95 | C_19_H_34_O_2_ | 294.2559 | 293.2481 | -0.5 | [M-CH_3_] 279.2324[C_18_H_31_O_2_]^-^, [M-OCH­_3_-C_11_H_21_] 109.0652[C_7_H_9_O]^-^ | -H | Methyl linoleate | RS | esters |
| 193 | 21.20 | C_16_H_32_O_2_ | 256.2402 | 255.2327 | -0.3 | [M-CH_3_] 241.2169[C_15_H_29_O_2_]^-^, [M-C_4_H_9_] 199.1697[C_12_H_23_O_2_]^-^, [M-C_2_H_5_] 227.2008[C_14_H_27_O_2_]^-^, [M-OH-CH_3_] 225.2213[C_15_H_29_O]^-^ | -H | Palmitic acid | CH, BZ  FL, GQ  GZ, HQ  RS | organic acids |
| 194 | 21.41 | C_9_H_16_O_3_ | 172.1099 | 171.1021 | -0.6 | [M-2CH_3_] 139.0393[C_7_H_7_O_3_]^-^, [M-C_4_H_10_] 113.0236[C_5_H_5_O_3_]^-^, [M-C_2_H_5_-CH_3_] 125.0236[C_6_H_5_O_3_]^-^, [M-CH_3_-C_4_H_7_] 101.0243[C_4_H_5_O_3_]^-^ | -H | Ethyl pivaloylacetate | BZ | esters |
| 195 | 21.41 | C_18_H_34_O_2_ | 282.2559 | 281.2484 | -0.2 | [M-OH-C_10_H_19_] 125.0965[C_8_H_13_O]^-^, [M-C_2_H_5_] 253.2163[C_16_H_29_O_2_]^-^, [M-CH_3_] 267.2321[C_17_H_31_O_2_]^-^, [M-CH_3_-OH] 251.2371[C_17_H_31_O]^-^ | -H, +HCOO | Oleic acid | CH, GZ  MH | organic acids |
| 196 | 21.41 | C_4_H_6_O_4_ | 118.0266 | 117.0180 | -1.3 | [M-OH] 101.9244[C_4_H_5_O_3_]^-^ | -H | succinic acid | DH | organic acids |
| 197 | 22.02 | C_17_H_34_O_2_ | 270.2559 | 269.2480 | -0.6 | [M-OCH_3_-C_7_H_15_] 141.1276[C_9_H_17_O_2_]^-^, [M-CH_3_-C_4_H_9_] 195.1381[C_12_H_19_O_2_]^-^, [M-OCH_3_-C_8_H_17_] 127.1118[C_8_H_15_O]^-^, [M-C_7_H_15_] 155.1067[C_9_H_15_O_2_]^-^ | -H | Methyl palmitate | HQ, GQ  MH, RS | esters |
| 198 | 22.72 | C_18_H_36_O_2_ | 284.2715 | 283.2640 | -0.3 | [M-C_3_H_7_] 241.2169[C_15_H_29_O_2_]^-^, [M-C_2_H_5_] 255.2324[C_16_H_31_O_2_]^-^, [M-C_4_H_9_] 227.2008[C_14_H_27_O_2_]^-^, [M-OCH_3_-C_9_H_19_] 125.0964[C_8_H_13_O]^-^ | -H | Methyl margarate | HQ, RS | esters |
| 199 | 25.86 | C_7_H_6_N_4_S | 178.0313 | 223.0282 | -1.4 | [M-CS] 135.8942[C_6_H_6_N_4_]^-^ | +HCOO | 1-Phenyltetrazole-5-thiol | MH | others |

* CH represents *B. chinense*, HQ represents *S. baicalensis*, MH represents *E. sinica*, GZ represents *C. cassia*, DH represents *R. glutinosa*, FL represents *S. glabra*, BZ represents *A. macrocephala*, GQ represents *L. chinense*, RS represents *P. ginseng* and GC represents *G. uralensis*.

**Supplementary** **Table 2.** The docking score of the core target binding to the related compound

| target | compound | docking score | target | compound | docking score |
| --- | --- | --- | --- | --- | --- |
| Mapk1 | Nicotiflorin | -9.911 | Pik3ca | Naringenin | -8.478 |
| Mapk1 | Cistanoside A | -8.506 | Pik3ca | Rivularin | -7.543 |
| Pik3cg | Narcissoside | -7.697 | Pik3ca | Vestitol | -7.365 |
| Mapk1 | Genkwanin | -7.652 | Pik3ca | Dihydrooroxylin | -7.288 |
| Mapk1 | Hesperidin | -7.6 | Pik3ca | Neocnidilide | -7.146 |
| Mapk1 | Dihydrobaicalin | -7.594 | Pik3ca | Glabranin | -6.986 |
| Mapk1 | Isorhamnetin | -7.553 | Pik3ca | Valerophenone | -6.497 |
| Mapk1 | Diosmetin | -7.425 | Pik3ca | Caffeic acid | -5.893 |
| Mapk1 | Picein | -7.423 | Pik3ca | Elemicin | -5.885 |
| Mapk1 | Isomartynoside | -7.23 | Pik3ca | Undecan-4-olide | -5.155 |
| Mapk1 | Acacetin | -7.213 | Pik3ca | Monobutyl phthalate | -4.764 |
| Mapk1 | Forsythiaside | -7.075 | Pik3ca | Butyl octyl phthalate | -4.485 |
| Mapk1 | Cistanoside F | -6.948 | Pik3ca | Methyl palmitoleate | -0.857 |
| Mapk1 | Acteoside | -6.929 | Pik3ca | 2-Tetradecanone | -0.02 |
| Mapk1 | Naringin | -6.571 | Pik3cg | Diosmetin | -9.034 |
| Mapk1 | L-Adenosine | -6.512 | Pik3cg | Viscidulin Ⅲ | -8.661 |
| Mapk1 | Ginsenoside rf | -6.464 | Pik3cg | Apigenin | -8.516 |
| Mapk1 | Glepidotin B | -6.356 | Pik3cg | Scutellarein | -8.403 |
| Mapk1 | Pectolinarigenin | -6.195 | Pik3cg | Isorhamnetin | -8.061 |
| Mapk1 | Caffeic acid | -5.872 | Pik3cg | Acacetin | -7.917 |
| Mapk1 | Glycyroside | -5.563 | Pik3cg | Quercetin | -7.914 |
| Mapk1 | Monobutyl phthalate | -5.376 | Pik3cg | Narcissoside | -7.852 |
| Mapk1 | Vulgarin | -5.053 | Pik3cg | Jaranol | -7.754 |
| Mapk1 | malkangunin | -4.929 | Pik3cg | Isomartynoside | -7.75 |
| Mapk1 | Ginsenoside rh1 | -4.174 | Pik3cg | Genkwanin | -7.379 |
| Mapk1 | Linoleic | -1.656 | Pik3cg | Dihydrooroxylin | -7.306 |
| Mapk1 | Methyl 9-oxononanoate | -1.192 | Pik3cg | Pectolinarigenin | -7.257 |
| Mapk1 | linolenic acid | -0.967 | Pik3cg | Baicalein | -7.24 |
| Mapk1 | Palmitic acid | -0.145 | Pik3cg | Hesperidin | -7.102 |
| Mapk1 | Myristic acid | 0.158 | Pik3cg | Kaempferol | -7.026 |
| Mapk1 | Saikosaponin a | — | Pik3cg | Rivularin | -7.019 |
| Mapk1 | Saikosaponin K | — | Pik3cg | Liquiritin apioside | -7.001 |
| Mapk1 | Saikosaponin f | — | Pik3cg | Herbacetin | -6.752 |
| Mapk1 | Licorice-saponin K2 | — | Pik3cg | Licoricidin | -6.613 |
| Mapk1 | Ginsenoside La | — | Pik3cg | Wogonin | -6.508 |
| Mapk3 | Diosmetin | -7.898 | Pik3cg | Glepidotin B | -6.493 |
| Mapk3 | Acacetin | -7.876 | Pik3cg | Naringin | -6.304 |
| Mapk3 | Apigenin | -7.625 | Pik3cg | Vestitol | -6.277 |
| Mapk3 | Pectolinarigenin | -7.604 | Pik3cg | Glabranin | -6.275 |
| Mapk3 | Scutellarein | -7.395 | Pik3cg | Skullcapflavone II | -6.246 |
| Mapk3 | Kaempferol | -7.37 | Pik3cg | Echinatin | -6.097 |
| Mapk3 | Baicalein | -7.063 | Pik3cg | Ethyl methoxycinnamate | -5.966 |
| Mapk3 | Herbacetin | -7.04 | Pik3cg | Gomisin B | -5.008 |
| Mapk3 | Genkwanin | -6.947 | Pik3cg | Undecan-4-olide | -4.419 |
| Mapk3 | Linoleic | -1.493 | Pik3cg | Ginsenoside rh1 | -4.413 |
| Mapk3 | zoomaric acid | -1.488 | Pik3cg | Amylbenzene | -4.008 |
| Mapk3 | linolenic acid | -1.444 | Pik3cg | Poricoic acid A | -3.537 |
| Mapk3 | Oleic acid | -0.194 | Pik3cg | 18β-Glycyrrhetinic Acid | -3.165 |
| Akt1 | Genkwanin | -4.959 | Pik3cg | pachymic acid | -2.717 |
| Akt1 | Viscidulin Ⅲ | -4.502 | Pik3cg | Mairin | -2.672 |
| Akt1 | Quercetin | -4.484 | Pik3cg | Methyl palmitoleate | -0.314 |
| Akt1 | Fraxetin | -4.459 | Pik3cg | 2-Tetradecanone | 0.567 |
| Akt1 | Kaempferol | -4.456 | Pik3cg | Saikosaponin a | — |
| Akt1 | Scutellarein | -4.402 | Pik3cg | Saikosaponin K | — |
| Akt1 | Herbacetin | -4.342 | Pik3cg | Saikosaponin f | — |
| Akt1 | Vulgarin | -4.306 | Pik3cg | Saikosaponin e | — |
| Akt1 | Eriodictyol | -4.21 | Pik3cg | Licorice-saponin G2 | — |
| Akt1 | Isorhamnetin | -4.193 | Pik3cg | Licorice-saponin K2 | — |
| Akt1 | Apigenin | -4.186 | Pik3cg | Glycyrrhizic acid | — |
| Akt1 | Diosmetin | -4.08 | Pik3cg | Ginsenoside Ro | — |
| Akt1 | Pectolinarigenin | -4.07 | Pik3cg | notoginsenoside R2 | — |
| Akt1 | Acacetin | -4.022 | Pik3cg | Maslinic acid | — |
| Akt1 | Baicalein | -3.692 | Pik3cg | Ginsenoside rf | — |
| Akt1 | Jaranol | -3.488 | Raf1 | Formononetin | -5.531 |
| Akt1 | Wogonin | -3.218 | Raf1 | Vestitol | -5.477 |
| Akt1 | 2-Tetradecanone | 2.463 | Raf1 | Glypallichalcone | -5.177 |
| Akt1 | Methyl laurate | 2.579 | Raf1 | Echinatin | -4.703 |
| Map2k1 | Diosmetin | -8.34 | Raf1 | Glabranin | -4.48 |
| Map2k1 | Acacetin | -7.876 | Raf1 | Undecan-4-olide | -3.637 |
| Map2k1 | Genkwanin | -7.587 | Raf1 | Methyl octylate | -2.265 |
| Map2k1 | Glypallichalcone | -6.935 | Raf1 | Methyl palmitoleate | -0.094 |
| Map2k1 | Vestitol | -6.905 | Raf1 | 2-Tetradecanone | 0.73 |
| Map2k1 | Physcion | -6.5 | Prkca | Neocnidilide | -6.14 |
| Map2k1 | Isorhamnetin | -6.238 | Prkca | Vulgarin | -5.832 |
| Map2k1 | Vulgarin | -4.671 | Prkca | Poricoic acid B | -4.345 |


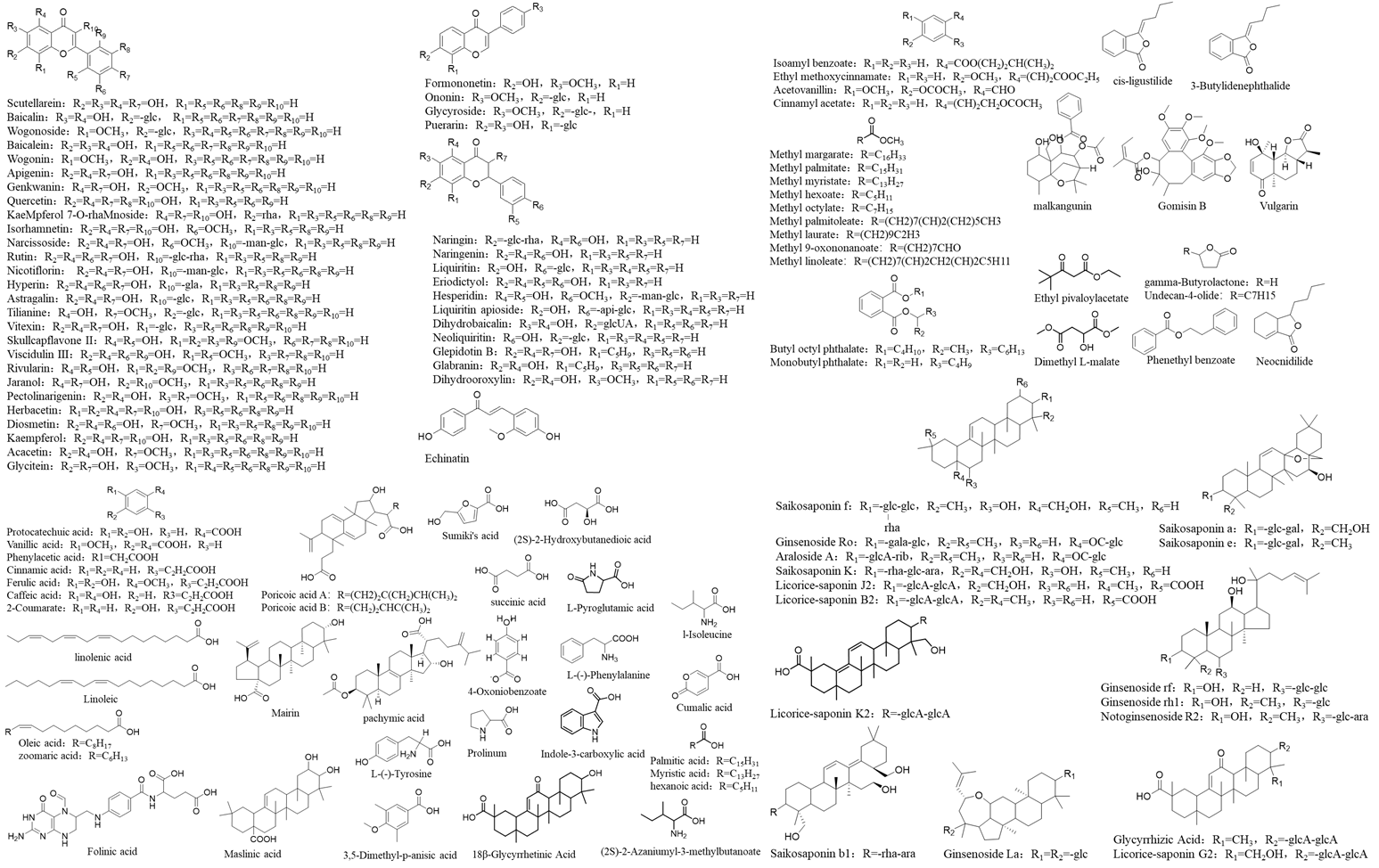

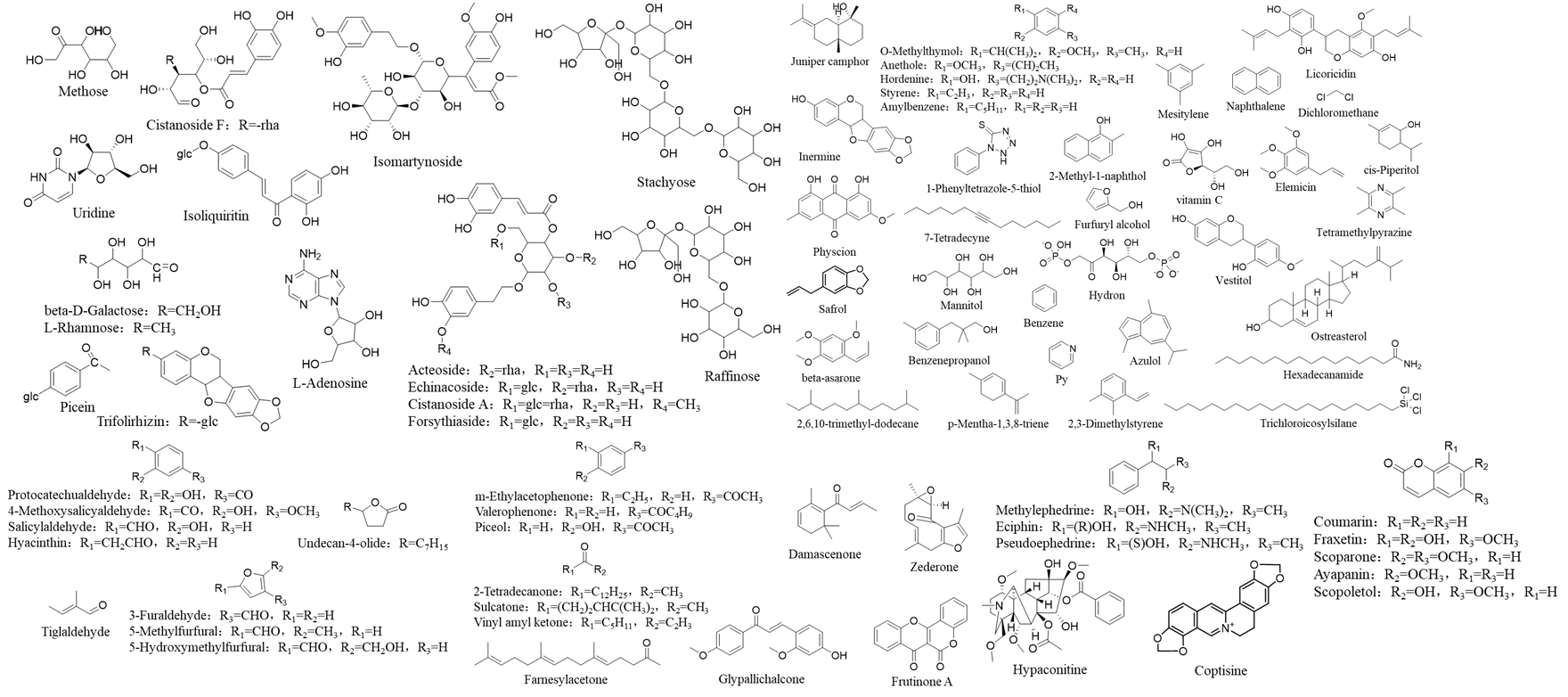


**Supplementary Figure 1.** Structural characterization of all detected components in XHP.
